# Mechanochemical preparation of strongly emissive monosubstituted triarylphosphane gold(i) compounds activated by hydrogen bonding driven aggregations†

**DOI:** 10.1039/d3ra03681b

**Published:** 2023-08-24

**Authors:** Lorenzo Luciani, Nicola Sargentoni, Claudia Graiff, Miguel Monge, María Rodríguez-Castillo, José M. López-de-Luzuriaga, Rossana Galassi

**Affiliations:** a University of Camerino, School of Science and Technology, Chemistry Division c/o ChIP, Via Madonna delle Carceri, 10 Camerino I-62032 Italy rossana.galassi@unicam.it; b Department of Chemistry, Life Sciences and Environmental Sustainability, Università degli Studi di Parma Parco Area delle Scienze 17/A Parma I-43124 Italy; c Universidad de La Rioja, Departamento de Química, Área de Química Inorgánica, Centro de Investigación en Síntesis Química, Complejo Científico-Tecnológico Madre de Dios, 53 26004 Logroño La Rioja Spain

## Abstract

Gold(i) triarylphosphane compounds are a well-known class of coordination compounds displaying from mild to strong emissive properties. Mechanochemical approaches to the preparation, spectroscopic characterization, X-ray diffraction structural determination, and photophysical studies of green emissive neutral linear monophosphane or neutral pseudo-T-shaped or cationic bis-phosphane gold(i) compounds, are herein discussed. The mechanochemical approach to the preparation of gold(i) derivatives was particularly successful for ligands bearing the carboxylic group, while the preparation with esterified ligands yields better results with solvent-mediated methods. The introduction of carboxyl or ester substituents in one aryl group favors the ligand-centered emissions. The analysis of the origin of the emissions was elucidated on the basis of DFT calculations, addressing the emissive behavior to ligand-centered excited states, strongly affected by supramolecular reversible hydrogen bonding aggregation. The study indicates that the ligand with the carboxylic group is particularly suitable for the mechanochemical preparation of emissive gold(i) complexes for material science applications.

## Abstract 2

LAG mechanochemical methods are applied for the preparation of luminescent di- and tri-coordinated gold(i) derivatives. Among them, a distorted T shaped bis-phosphane gold(i) chloride compound strongly emits upon self-aggregation by hydrogen bonding.

## Graphical abstract

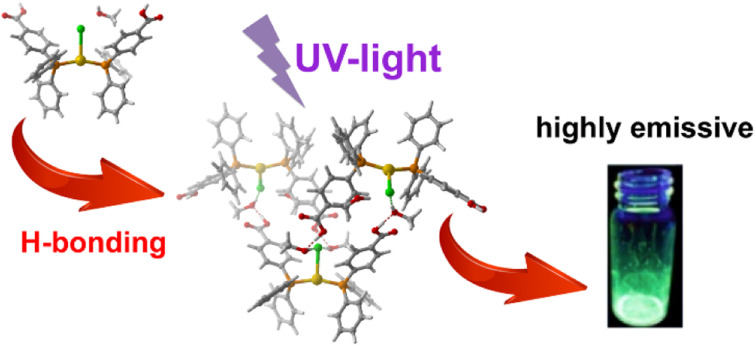

## Introduction

Phosphane compounds of Au(i) have been known since the early age of the coordination chemistry of this metal.^1^ The interest in these compounds arises from their applications in the fields of bioinorganic chemistry as an anticancer or theranostic agents, or for bioimaging,^2–5^ in homogeneous catalysis^6^ and in materials science.^7,8^ The application of this class of gold(i) compounds in material science is also related to the design of luminescent transition metal-based materials featuring low-lying triplet excited states and phosphorescence.^9^ The photophysical properties of bis-phosphane gold(i) complexes have been largely investigated since the first report in 1989 of the photoluminescence for [Au_2_(μ-dppm)_2_]^2+^ (with dppm = bis(diphenylphosphino)methane) that was independently reported by Fackler^10^ and Che.^11^ In these reports the long-living phosphorescence recorded at 593 nm was attributed to the Au⋯Au bonded excited state. Moreover, the luminescence of mono-, bis- or tris-(triphenylphosphane)gold(i) compounds was attributed to the geometry around the metal center, with the tri-coordinated phosphine gold(i) complexes being the most emissive.^10,12,13^ Conversely, the bi-coordinated phosphane gold(i) complexes are weakly emissive unless at cryogenic temperatures in the solid state, or closed shell d^10^–d^10^ bonding occur in solution; for these latter, any distortion from the linear coordination triggers rather strong emissions.^14^ The luminescent properties of phosphane gold(i) complexes are appealing for sensing, theranostic, and bioimaging applications and, not least, for optoelectronic devices; a promising attempt to apply complexes with phosphane and d^10^ closed shell metals in the fabrication of OLED prototypes were reported in 2011;^15^ the gold complexes performance was outlined by a *Φ*_max_ = 35% in solid state and electroluminescence efficiency (EL) equal to 6.6%. The analysis of the photophysical properties has been largely improved with the occurrence of theoretical studies and computational processing, but the preparation of pure luminescent materials is still largely related to classic solvent-mediated synthesis.^10,16^ We are currently dealing with increased environmental awareness, hence technical efforts and grants are devoted to finding sustainable material preparations. Among the possible strategies, the mechanochemical methods have gained a growing interest due to the low environmental impact of the preparations and the direct economic involvements that might result from a large-scale application.^17^

**Scheme 1:**
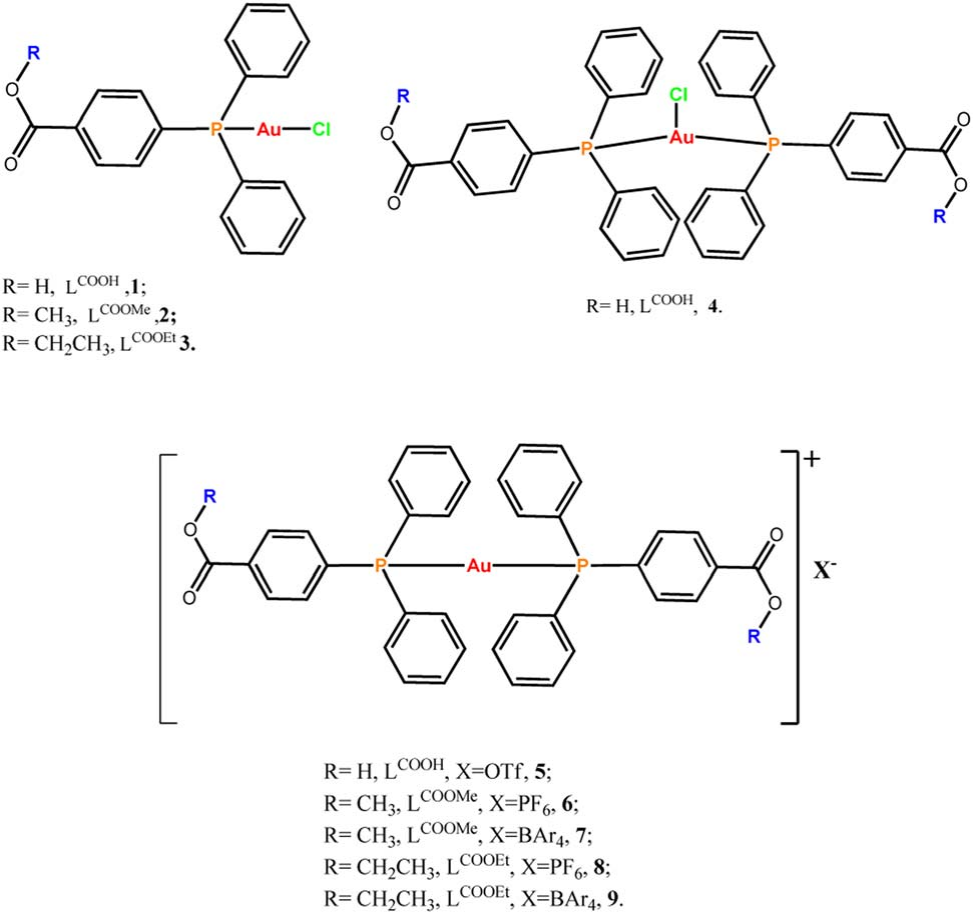
List of the compounds discussed in this work.

Mechanochemical reactions involving Au(iii) compounds and resulting in the formation of Au–C bonds have been recently reported,^18^ as well as for Au(i) compounds,^18–21^ while the formation of AuOH derivatives starting from LAuCl and KOH (where L is the JohnPhos ligand) were observed by C_6_D_6_ adding grinding.^22^ In the literature, few cases of mechanochemical preparations of luminescent phosphane gold(i) compounds are reported; mainly they consist of ball milling or mortar grindings.^23,24^ The scarcity of mechanochemical applications in these preparations may be since phosphane ligands and gold(i) centers may yield neutral or cationic derivatives displaying, for example, P–Au–X (with X = counterion) or P–Au–P^+^ X^−^ environments, or to derivatives where the ligand to metal ratio is even larger;^16,25^ indeed, the formation of neutral compounds with three or four-coordinated gold(i) centers may be also observed, depending on the coordinative ability of the counterion X^26–28^ and the donor properties of the phosphane ligand.^29–31^ In this work, the mechanochemical approach has been applied to the synthesis of luminescent mononuclear mono- or bis-phosphine gold(i) compounds by employing triaryl phosphane ligands having a carboxylic or the related methyl or ethyl ester group (see Scheme 1); the preparations were led by LAG (Liquid Assisted Grinding) in a mortar at room temperature. The solvent-mediated and mechanochemical preparations were compared, and the results converge in the formation of the same products by both methods (compounds 1, 2, 3, and 4), but also to unexplainable failures (compounds 6 and 8). The most successful preparations in terms of yield and purity were achieved with the 4-diphenylphosphanyl-benzoic acid as the ligand. It is likely to attribute the formation of the bis-phosphane gold(i) chloride complex 4 to the presence of the carboxylic group, whose detailed structural and emissive properties studies, along with the computational modelling, highlighted the adoption of the tri-coordinated T-shaped geometry at the metal centre and a further trimeric oligomerization in the solid state packing, held by hydrogen bonding aggregations.

## Results and discussion

### Synthetic procedures and characterizations

**Fig. 1:**
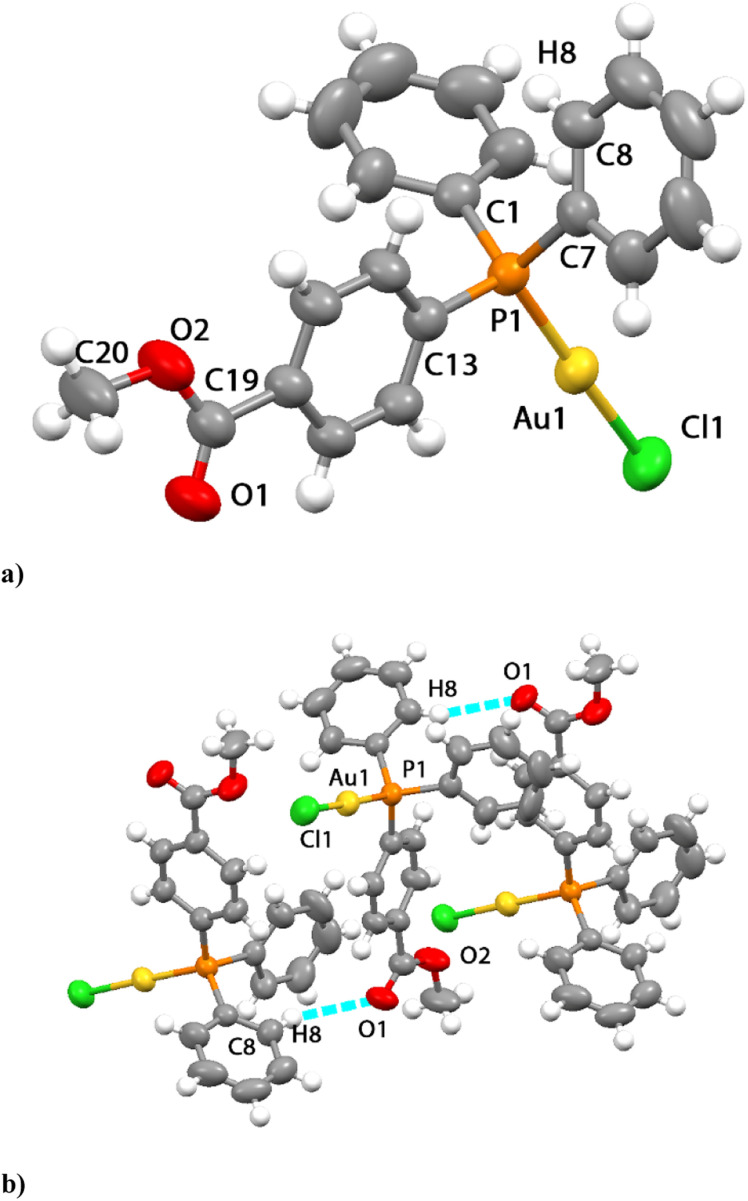
ORTEP of cell units obtained by the X-ray single crystal structure diffraction for compound 2 showing (a) the independent unit (50% probability level for the displacement ellipsoids), and (b) the hydrogen bonding occurring in the packing of the unit cell.

The introduction of a carboxylic or an ester group in the *para* position of one phenyl of the Ph_3_P moiety modifies the donor properties of the ligand,^32^ while it is negligible the effect of the methyl or ethyl group in the ester. The ligands L^COOH^, L^COOMe^, or L^COOEt^ (corresponding to the 4-diphenylphosphine-benzoic acid, L^COOH^, and the relative methoxy or ethoxy esters, L^COOMe^ and L^COOEt^, respectively) were used for the preparation of the corresponding monophosphane gold(i) chloride compounds and the bis-phosphane gold(i) compounds. The electronic donor properties of the three ligands are similar, in fact, the ^31^P NMR chemical shift of L^COOH^ is slightly less negative than those of L^COOMe^ and L^COOEt^ (see Table 1S in ESI†). Interestingly, the ligand L^COOH^ is both a Hydrogen Bonding (HB)-donor and an HB-acceptor ligand leading to complemental intermolecular hydrogen bonds in the crystal structure of both L^COOH^AuCl (1)^33^ and (L^COOH^)_2_AuCl (4) (Fig. 1). The LAuCl compounds 1–3 were isolated in good yields, despite the concomitant precipitation of the related bis-phosphane-gold(i) compounds to some extent, which were removed by selective solvent extractions.

Beyond solvent-mediated methods, L^COOH^AuCl (1) and (L^COOH^)_2_AuCl (4) were prepared by mechanochemical methods in rather high yields (Table 1, 70% and 84% of yield, respectively), according to the 1 : 1 and 2 : 1 mole ratio between the ligand and the Me_2_SAuCl, respectively. The stepwise or the one-pot addition of the 2 moles of L^COOH^ to the Me_2_SAuCl do not affect significantly the yield (+∼10% for the latter). Despite the donor skill similarity, the same preparative method was unsuccessful to obtain (L^COOMe^)_2_AuCl or (L^COOEt^)_2_AuCl complexes; for these latter, mixtures of mono and bis-phosphane products were obtained, affording to the exclusive isolation of the mono-phosphane L^COOMe^AuCl (2) or L^COOEt^AuCl (3) upon crystallization. Therefore, the corresponding bis-phosphane gold(i) compounds were prepared according to solvent-mediated methods. Given the non-innocent behavior of the chloride ions in the photoemissive properties of gold(i) compounds, a series of L_2_AuX compounds, 5–9, with X = OTf^−^ (trifluoromethanesulfonate), PF_6_^−^ (hexafluorophosphate) or the Brookhart anion, BAr_4_ (tetrakis[(3,5-trifluoromethyl)phenyl]borate) were prepared by metathesis reactions with the corresponding silver salts. Compound 5 was also prepared to compare the stability of the bis-phosphane-gold cation relatively to anions of different nature, such as the triflate and the hexafluorophosphate, with this latter resulting prone to be hydrolysed in the presence of water (*vide infra*). The coordination of the chloride in compounds 1–3 was evidenced in the infrared spectra in the range 400–200 cm^−1^ and confirmed by X-ray crystal structure determinations. The IR spectra of compounds 1–3 display the typical bands for the Au–Cl stretching vibrational modes at 340 and 332 for 1, at 332 cm^−1^, and at 326 cm^−1^ for 2, at 332 and at 327 for 3 (see Fig. 1S–3S in ESI†). In compound 4, the different strength of the Au–Cl bond evidenced by the long Au–Cl bond length (*vide infra*),^33^ in addition to a change of the coordination geometry at the metal centre made difficult the assignment of the band; the IR spectrum of 4 does not exhibit the band centred at 330 cm^−1^, but a band is observed at 227 cm^−1^ that might be attributed to the vibrational mode of the Au–Cl bond stretching, by comparing it with that reported for the (PPh_3_)_2_AuCl 0.5 benzene at 221 cm^−1^ (Fig. 1S†).^34^ However, by analysing data reported in the literature, this attribution seems to be rather controversial, as most T-shaped P–Au(Cl)–P structures do not report IR data for the Au–Cl bond.^25,35,36^ Similarly to compound 4, the T-shaped structure of Au[(C_6_H_5_)_2_PCH_2_N(C_6_H_5_)_2_]_2_Cl displays an AuCl bond length rather long (2.951 Å), and in the far IR spectrum no bands attributable to the Au–Cl were assigned, arguing this lack of bands as an effect of the ionic/covalent character of this bond.^28^ At the ionic limit, as expected, the Au–Cl absorptions disappear in the IR spectra of 5–9. Beyond the Au–Cl vibrational modes, no large shifts in other bands are observed, in fact, the coordination of the metal center does not introduce significant changes in the mode of carbonyl stretching of the ligands, except for the blueshifts of 15 cm^−1^ and 8 cm^−1^ for the formation of bis-phosphine 7 and 9, respectively, whose cationic nature was ensured by the least coordinating anion in this series of compounds (see Table 1S in the ESI†).

**Table 1:** Reactions yields (%) of the mechanochemical and solvent mediated preparations of compounds 1–4, and of their solvent mediated metathesis reactions^a^

Compound	Mechanochemical	Solvent-mediated
1	70	68^b^
2	85	92
3	nr	94%
4	84	98^c^/82^d^
5	nr	76
6	nr	73
7	nr	45
8	nr	81
9	nr	42

a nr = not reported.

b Parameter taken from literature^30^.

c Yields relative to method A (see Experimental part).

d And to method B (see Experimental part).

### Solution studies

Solution studies were led by ^31^P NMR spectroscopy and ESI MS. For all the compounds 1–9 the formation of the cationic bis-phosphane in the ionization source is a common result in the ESI spectra, underlining the well-known higher stability of this structural motif than that of the monophosphane derivative. For a better evaluation of the ^31^P NMR data, the difference of the chemical shifts between the complexes and those of the starting ligands (*Δ*_ppm_, chemical shifts variation from the complex to the free ligand) for compounds 1–9 were reported in Table 1S in ESI.† The P atoms in the monophosphane-gold(i)-chloride compounds are more shielded than those of the corresponding bis-phosphane gold(i) compounds, displaying *Δ*_ppm_ ranging between +38 and +50 ppm.^37^ The ^31^P NMR signals of mono-phosphane complexes 1–3 are sharp at room temperature, instead most of the bis-phosphane complexes exhibit broad signals, likely indicating a dynamic behaviour of these latter in solution. The Full Width at Half Maximum (FWHM) goes from less than ten Hertz for complex 8 (10 Hz) or 9 (2 Hz), to hundreds of Hertz for complex 6 (160 Hz) (see Table 1S in the ESI†). The dynamic behaviour of bis-phosphine compounds 4–9 can be attributed to different counterion coordination options,^38,39^ ranging from the ionic to the three coordinate limits according to the following equilibrium [(PR_3_)_2_AuCl] ⇄ [(PR_3_)_2_Au]^+^ + Cl^−^, rather than to equilibria leading to the formation of tris- or tetra-phosphine compounds, since for the latter the thermodynamic constants of formation are generally very small. As quoted in the literature,^40^ a trend of binding ability of the anions can be depicted from the analysis of FWHM of compounds 4–9 as follows: Cl^−^ > OTf^−^ > PF_6_^−^ > BAr_4_^−^. Interestingly, for the case of the compound, (L^COOMe^)_2_AuPF_6_ (6) the ^31^P NMR evidenced the complete hydrolysis of the anion and the formation of the PO_2_F_2_^−^ anion (triplet centred at −18.38 ppm, see Fig. 5S in the ESI†). Among the series of bis-phosphane-gold(i) complexes 4–9, the most shielded ^31^P NMR signal (36.65 ppm) and the highest value of FWHM (160 Hz) were recorded, indicating the fast equilibrium exchange between the [AuL_2_PO_2_F_2_] ⇄ [AuL_2_]^+^ + PO_2_F_2_^−^.^41^ The formation of the PO_2_F_2_^−^ was observed either using fresh silver or thallium hexafluorophosphate salts (the absence of the PO_2_F_2_^−^ anion in the initial salt was verified by ^31^P NMR) and by an accurate checking of the water content into the solvents. However, some adventitious water was presumably present in the dried AgPF_6_ salts or in the deuterated solvent solution. The hydrolysis occurred only in solution, as the IR spectrum of the solid (L^COOMe^)_2_AuPF_6_ (6) showed the expected peaks for the PF_6_^−^ anion (Fig. 6S, see ESI†) due to the IR active P–F bands at 830 cm^−1^ and 748 cm^−1^. So far, few cases of the hydrolysis of PF_6_^−^ anion are reported in the literature with palladium complexes.^41^

### Crystallography

**Fig. 2:**
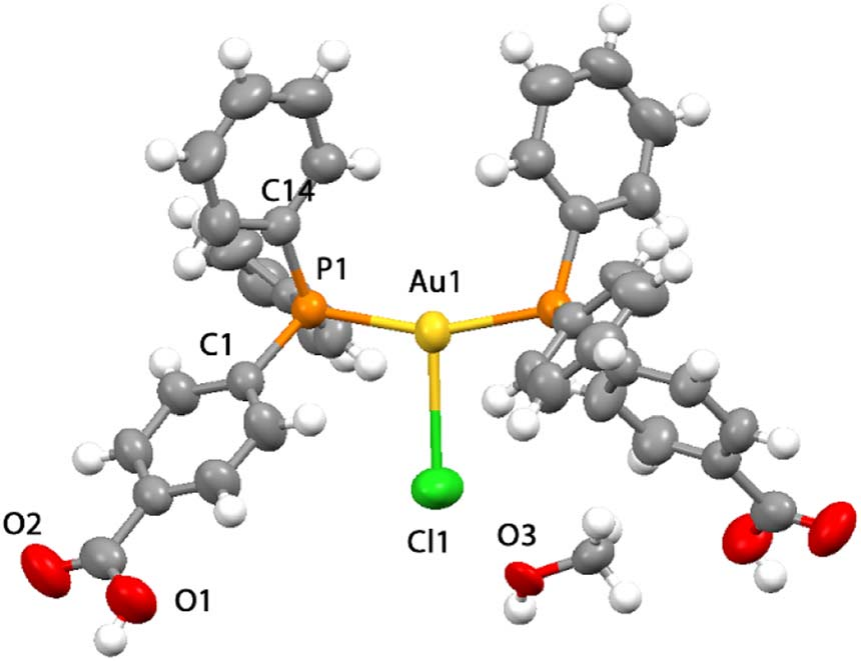
View of the crystal structure obtained by the X-ray diffraction for compound 4 with a molecule of methanol (50% probability level for the displacement ellipsoids).

**Fig. 3:**
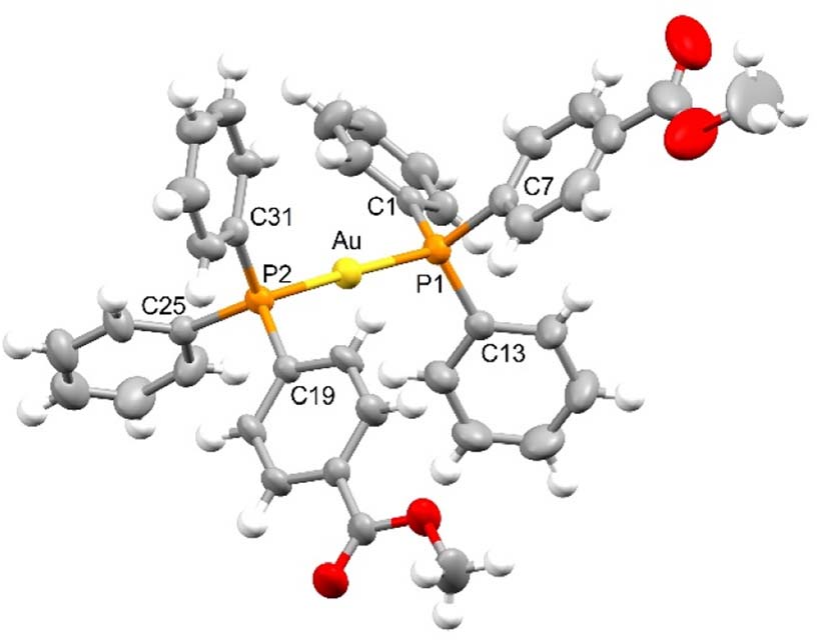
View of the cationic complex [bis(4-diphenylphosphanyl-methylbenzoate)gold(i)] (7) (30% probability level for the displacement ellipsoids). Brookhart anion has been omitted for clarity.

Crystal data for compounds 2, 4, and 7 are reported in the ESI,† while a selection of bond distances and angles is reported in Table 2. The X-ray crystal structure of compound 1 was solved and it was consistent with that overlapping with that already published by Mohr *et al.*,^33^ while that of compound 2 is shown in Fig. 1, consisting of a neutral monophosphane compound with a linear P–Au–Cl framework. The molecular structure of the neutral compound 4 is shown in Fig. 2; it displays a bis-phosphane moiety with the chloride coordinated to the gold center in a distorted T-shaped arrangement; finally, the molecular structure of compound 7 is shown in Fig. 3; this latter is a bis-phosphane cation with a linear P–Au–P geometry, with the Brookhart anion not coordinated. Comparing the structures of 1 (ref. 33) and 2, besides the linear coordination of the P–Au–Cl, that is displayed both for 1 and 2 with angles of 177.72 and 177.35, respectively, shorter P–Au at 2.225 Å and 2.270 Å for Au–Cl bond distances were observed for compound 1. Interestingly, in compound 1, the presence of the carboxyl group in the ligand activates the coupling of pairs of molecules by hydrogen bonds, contrary to what is observed in compound 2 which contains the methyl ester ligand.

**Table 2:** Selected bond distances and bond angles for compounds 1, 2, 4, and 7

Compounds	1^a^	2	4	4	7
P–Au bond length	2.225(1)	2.231(1)	2.306(1)	2.231(1)	2.303(2)
Au–Cl bond length	2.270(2)	2.284(2)	2.857(2)	2.857(2)	—
P–Au–Cl bond angle	177.72(6)	177.35(5)	100.42(2)	100.44 (3)	—
P–Au–P bond angle	—	—	159.15(5)	159.12(6)	177.82(6)
C–H⋯OH-bond length	—	2.699(3.459)^b^	—	—	—
O–H⋯OH-bond length	—	—	1.828(2.613)^b^	1.813(2.622)^b^	—

a Parameters taken from literature;^33^.

b Donor (D)–acceptor (A) distance.

The structure of compound 4 contains a tri-coordinated geometrical arrangement at the metal center that is unusual for gold phosphane compounds, as most bis-phosphane compounds are ionic with the counterion far apart from the metal centers.^25,42,43^ The length of the Au–Cl bond was found to be 2.858 Å which, although at the long limit, is shorter than the sum of the van der Waals radii indicating the coordination of the chloride ion to the gold atom.^44^ The Au–Cl bond length in 4 is longer than what was observed for [(Ph_3_)_2_PAuCl] in two different reports at 2.526 Å (ref. 34) or at 2.533 Å,^23^ but shorter than that observed for a similar three coordinated chloro-bis(diphenylphosphino-diphenylamino-methane-P)-gold(i) derivative with an Au–Cl at 2.952 Å.^26^ The molecular symmetry in 4 displays the molecule disposed on a crystallographic two-fold symmetry axis coincident with the metal–halogen bond and with one-half the molecule comprising the asymmetric unit (Fig. 2). The P–Au–P angle is ∼159° and both the P–Au–Cl angles are ∼100°, indicating a strongly distorted trigonal coordination approaching to a T-shaped geometry.

In the crystals of compound 7, cations of [bis(4-diphenylphosphanyl-methylbenzoate)gold(i)] and anions [tetrakis(3,5-bis(trifluoromethyl)phenyl)borate] are present and a linear P–Au–P geometry is found. The well-known Brookhart anion does not deserve any particular attention. Fig. 3 reports a view of the cationic complex and the atomic labelling scheme. The most important bond distances (Å) and angles (°) are Au–P1 2.3028(16), Au–P2 2.3031(15), C1–P1 1.803(6), C7–P1 1.821(7), C13–P1 1.801(6), C19–P2 1.816(6), C25–P2 1.807(6), C31–P2 1.798(6); P1–Au–P2 177.81(5). The gold atom is coordinated by the two ligands in a nearly linear environment through the phosphorus atoms. The P–Au–P bond angle is 177.81(5)°. The two P–Au bond distances are equal 2.303(2) Å and comparable with P–Au lengths reported in the literature for parent bis-phosphane gold(i) compounds.^37,45,46^ Noteworthy, in all the crystal structures herein presented no aurophilic interactions occur and the crystal packings are built up by van der Waals interactions or hydrogen bonding. As in example, in complex 7, the carbonyl moiety of the methylbenzoate unit is involved in short contact with a hydrogen atom of a phenyl ring of an adjacent cationic complex, allowing the formation of a dimeric system in which the two methylbenzoate groups are stacked at 3.8 Å. The steric hindrance of the triarylphosphine ligands^30,31^ and the presence of substituents skilled to accept and/or donate hydrogen bonding dominate over the occurrence of the well-known d^10^–d^10^ closed shell interactions, whose strength is comparable to that of the hydrogen bonding.

### Photophysical studies

Taking into account the three different coordination situations discussed (neutral linear; ionic linear; and pseudo-trigonal planar), we carried out an in-depth study of the photophysical behaviour of three representative examples of this kind of complexes, namely complexes 2, 4, and 7. The three complexes show similar characteristics in the diffuse reflectance absorption spectra (Fig. 7S in ESI†). All three show strong absorptions in the 200–375 nm range that are fairly featureless and that are likely to be assigned to allowed transitions located in the ligands, probably involving π–π* transitions between orbitals in the rings. Usually, when gold is involved in the transitions as metal-centred or charge transfer ones, the absorption should appear at lower energies.^47^ In the three cases, a complete absence of luminescence in deoxygenated acetonitrile solutions is observed, but they do show emissions at room temperature and at 77 K in the solid state. This result is interpreted in terms of equilibrium processes in solution that lead to different species in that media, or quenching processes promoted by the formation of exciplexes between complexes and solvent.

**Fig. 4:**
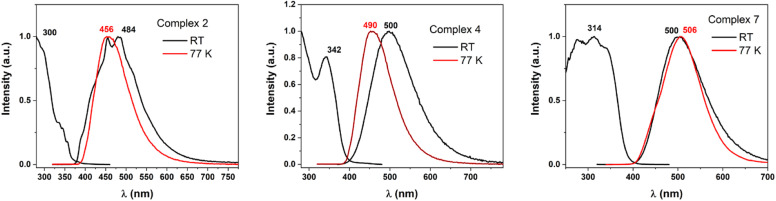
Excitation and emission spectra at RT (black lines) and emission spectra at 77 K (red lines) recorded for complexes 2, 4, and 7 in the solid state.

As it was commented, the complexes display weak to moderate luminescence when they are irradiated with UV light (Fig. 4). Complex 2 displays an almost non-perceptible luminescence at room temperature, which increases when the measurement is recorded at 77 K, by contrast, complexes 4 and 7 display easily visible luminescence to the human eye at room temperature which becomes bright at a low one. This brings to light the quantum yield measurements of the derivatives with values of 6 and 15.5% for complexes 4 and 7, but a modest 1.8% for 2, at room temperature, respectively. The emissions appear in the bluish-green zone in all cases. The emissive spectral data referred to compounds 2, 4, and 7 are reported in Table 3.

**Table 3:** Spectral data for compounds 2, 4, and 7

Labels	*λ*_em_ (*λ*_exc_), r. t.	*λ*_em_ (*λ*_exc_) 77 K	Yield %, r. t.	Lifetimes (μs)
2	469 (300)	446 (300)	1.8	43 (r. t.), 134 (77 K)
4	500 (342)	490 (325)	6	1.5 (r. t.), 75 (77 K)
7	500 (314)	506 (300)	15.5	13 (r. t.), 23 (77 K)

**Fig. 5:**
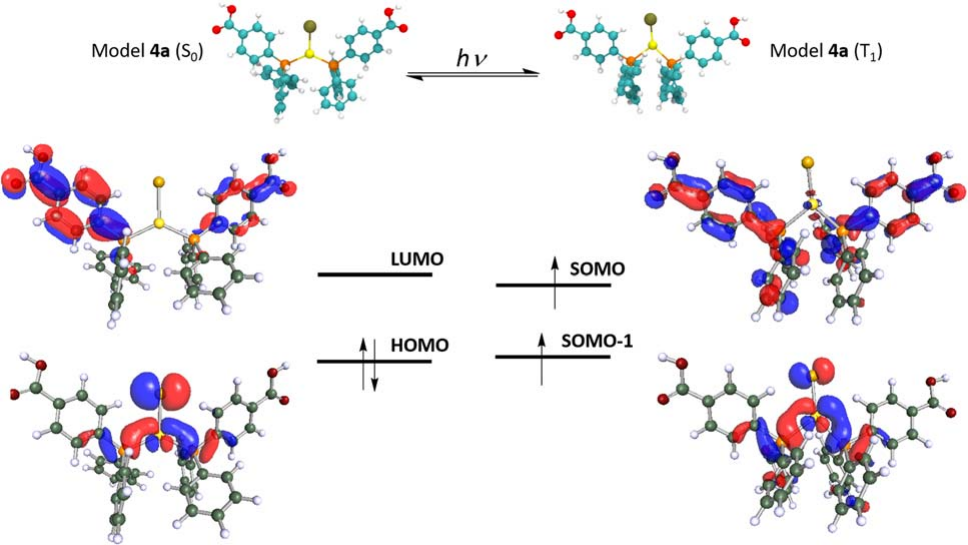
Optimized model system 4a in the ground and the phosphorescent (triplet) excited states (top). Frontier HOMO–LUMO (S_0_ state) and SOMO–SOMO-1 (T_1_ state) (bottom).

The long lifetimes found suggest forbidden transitions and, hence, phosphorescent processes, which are probably induced by the strong spin–orbit coupling caused by the presence of gold in the complexes that favours the intersystem crossing between the singlet and triplet states (see Fig. 5 and 8S in ESI†). The three complexes show similar absorption spectra and energetic zone for the emissions, although with slight variations; for instance, complex 2 and 7 presents the same ligand but display neutral linear and ionic linear molecular structures, respectively, moreover complex 4 exhibits pseudo-trigonal planar geometry and slightly different ligand, but similar emission of complex 7. Thus, we are prone to assign these emissions to arise from orbitals mostly ligand-based, perhaps with a small contribution of the gold centres that are lightly affected by the ligand coordination environments.

### DFT calculations

**Fig. 6:**
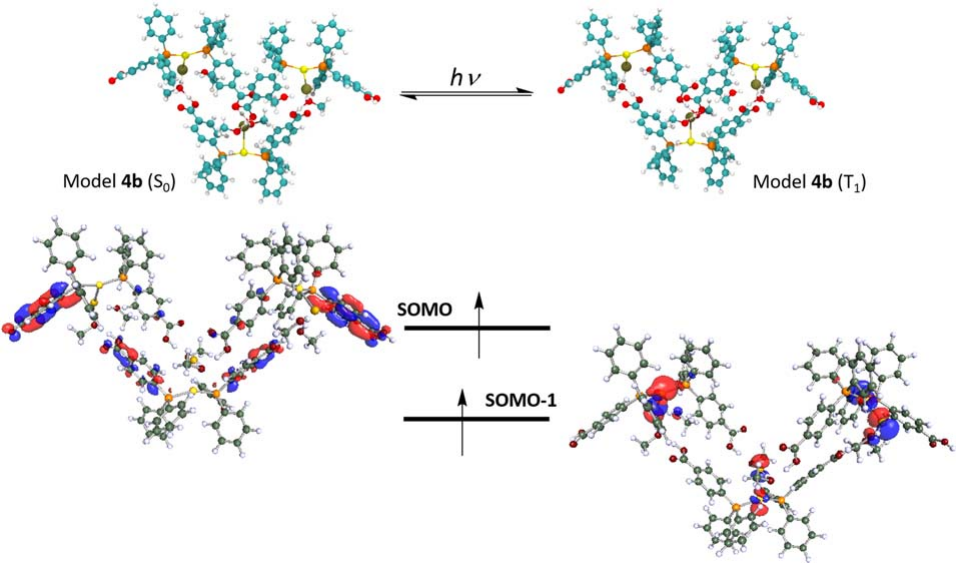
Optimized model system 4b in the ground and the phosphorescent (triplet) excited states (top). Frontier SOMO–SOMO-1 (T_1_ state) (bottom).

The interesting and less common structural and emissive properties found for complex 4 prompted us to carry out an in-depth DFT study. This complex resembles those of stoichiometry [Au(PPh_3_)_2_X] (X = Cl, Br, and I), previously studied by Omary and co-workers,^48^ for which a striking phosphorescent T_1_ excited state beyond a T-shape was proposed from an experimental and computational study. Indeed, the large distortion of the Au(i) environment found for the DFT-computed model [Au(PPh_3_)_2_Cl], especially in the Au–P distances and P–Au–P and Cl–Au–P angles would agree with electronic excitations involving the metal centres and their close environment. In the present case, we analysed computationally the optimized ground (S_0_) and lowest triplet excited state (T_1_) for two model systems of complex 4 (see Fig. 5 and 6). The first one consists of a mononuclear model [Au{(L^COOH^)}_2_Cl] (4a) and the second one is a trinuclear model system [Au{(L^COOH^)}_2_Cl]_3_ (4b) that represents the supramolecular arrangement of this molecule, in which the Cl atom is involved not only in the bonding to the Au(i) centre but also in H-bonding interactions with two methanol molecules. The presence of these solvent molecules in the supramolecular arrangement would provide a strong influence on the emissive excited state structure, which is interesting to account for from a computational viewpoint. Indeed, it is expected that the structural distortion found for model 4b would be closer to the experimental one displaying a more accurate description of the emissive behaviour of this compound. Table 4 displays the most relevant structural parameters found for complex 4 both experimentally and computationally. The optimization of the mononuclear model system [Au{(L^COOH^)}_2_Cl] (4a) in the ground state shows a similar trigonal disposition to that found in the X-ray diffraction experiment but the Au–Cl distance is shorter than the experimental one (2.466 Å *vs.* 2.858 Å), whereas the computed Au–P distances are slightly larger than the experimental ones (2.331 Å *vs.* 2.307 Å). In the same way, the Cl–Au–P angles are larger for the computed model (115.07, 115.30° *vs.* 100.44°), meanwhile, the calculated P–Au–P angle is shorter than the experimental one (129.63° *vs.* 159.12°). The rest of the distances and angles are similar. Therefore, although the optimized structure of model 4a in the ground state resembles the experimental one, we speculated about the possible influence of the supramolecular interactions both in the ground and in the excited state of complex 4. Indeed, if we have a look at the most important distances and angles computed for the supramolecular model [Au{(L^COOH^)}_2_Cl]_3_ (4b) in the ground state, we observe a very good match between these data and the experimental ones. This is observed especially for the calculated Au–Cl distance (2.788 Å *vs.* 2.858 Å); for the Au–P distances (2.322 Å *vs.* 2.307 Å); for the Cl–Au–P angles (99.75° *vs.* 100.44°); and for the P–Au–P angle (160.50° *vs.* 159.12°). It is also worth mentioning that the optimized structure of 4b also shows the presence of intermolecular O⋯H–O hydrogen bonding between the O atom of a neighbouring CO_2_H group and the methanol molecule.

**Table 4:** Most relevant distances (Angstroms) and angles (deg) for complex 4 and DFT-optimised models 4a and 4b in the ground state (S_0_) and lowest triplet excited state (T_1_)

	Exp.	4a S_0_	4a T_1_	4b S_0_	4b T_1_
Au–Cl	2.857	2.466	2.337	2.788	2.721
Au–P	2.306	2.331	2.367 and 2.408	2.322	2.330
C–P (CO_2_H–Ph)	1.824	1.825	1.803 and 1.805	1.814	1.805
C–C (CO_2_H–Ph)	1.379, 1.385–1.392 and 1.401	1.398, 1.402–1.410 and 1.411	1.391, 1.396–1.417 and 1.425	1.398, 1.401–1.409 and 1.410	1.397, 1.398–1.413 and 1.415
C–C (C–CO_2_H)	1.500	1.493	1.479	1.504	1.496
C–O (CO_2_H)	1.206 and 1.324	1.220 and 1.360	1.225 and 1.367	1.230 and 1.330	1.232 and 1.336
C–P (Ph)	1.820 and 1.825	1.825 and 1.827	1.802 and 1.807	1.820, 1.825	1.805
C–C (Ph)	1.356, 1.390–1.396 and 1.406	1.400, 1.404–1.407 and 1.413	1.398, 1.400–1.417 and 1.419	1.403, 1.404–1.410 and 1.411	1.403, 1.403–1.410 and 1.412
Cl–Au–P	100.42	115.07 and 115.30	122.23 and 139.18	99.75	100.57 and 101.68
P–Au–P	159.15	129.63	98.59	160.50	157.75
Au–P–C (CO_2_H–Ph)	116.37	118.73	108.35, 111.63	113.87	116.55 and 116.76
Au–P–C (Ph)	110.45 and 110.88	110.27 and 113.08	110.19 and 112.56	112.49 and 113.36	109.73 and 112.55
O⋯H–O	1.813	—	—	1.572	1.584

This result found in the optimization of a larger model system points to the need of taking into account, as much as possible, the supramolecular dispositions of the molecules in solid state and their corresponding intermolecular interactions. Indeed, this better computational description of the solid-state structure will also provide an improved description of the emissive excited state of complex 4 (*vide infra*). The optimization of model 4a in the T_1_ excited state displays a large structural distortion compared to the situation found in the S_0_ ground state. First, a shortening of the Au–Cl distance (2.466 Å *vs.* 2.337 Å) and an enlargement of the Au–P distances (2.331 Å *vs.* 2.367, 2.408 Å) are found. Second, a phosphine ligand is also noted (see data in Table 4). Therefore it is expected that charge transfer transitions involving MOs of the Au(i) environment and the phosphine ligand, specifically in the Ph–CO_2_H groups, would be responsible for the emissive behaviour of complex 4. We have confirmed this supposition by computing the HOMO and LUMO for 4a in the S_0_ state and the SOMO and SOMO-1 for 4a in the T_1_ state. Fig. 5 depicts these MOs and shows that the HOMO is located mostly in the Au(i) centre and the Cl ligand with some contribution from the P atoms. Meanwhile, the LUMO with a π* character is mostly placed in the Ph–CO_2_H groups of the phosphine ligands, suggesting that the origin of the emissive behaviour is a metal-chloride to phosphine ligand MXLCT charge transfer transition. Similarly, the T_1_ frontier orbitals SOMO and SOMO-1 display an analogous character, confirming that the emission from the lowest triplet excited state consists of the de-excitation of the electron from the π* SOMO to the metal-chloride SOMO-1. We have also computed the T_1_–S_0_ vertical emission for this model 4a in nm, clearly red-shifted with respect to the experimental one of 500 nm. Modification of the C–C and C–O distances in the Ph–CO_2_H groups consisting mostly of the distortion of the aromatic ring of the gas phase resulting in an emission at 675 red-shifted with respect to the experimental one of 500 nm.

As we have mentioned above, the better description of the experimental molecular arrangement found for model 4b also provides a more accurate description of the emissive properties. Regarding the optimization of 4b in the T_1_ state we obtain a similar pattern to the one described for 4a, but with less pronounced changes in the distances and angles. For example, the shortening of the Au–Cl distance (2.788 Å *vs.* 2.721 Å) and the enlargement of the Au–P distances (2.322 Å *vs.* 2.330 Å) are again observed. In addition, a slighter modification of the C–C and C–O distances in the Ph–CO_2_H groups are found (see data in Table 4). This tendency would be closer to the solid-state situation than that computed for the isolated mononuclear model 4a. In the case of model 4b, the frontier SOMO and SOMO-1 in the T_1_ excited state have been computed (see Fig. 6). The similar shape of these MOs confirm to that found for the same MOs in 4a confirms that the emissive behavior of complex 4 is due to the de-excitation of the electron from the π* SOMO to the metal-chloride SOMO-1, in a charge transfer process. The better representation of the emissive properties of complex 4 with this model allows the prediction of a computed emission value (vertical T_1_–S_0_ transition) of 487 nm (experimental 500 nm).

## Experimental

### Materials

4-Diphenylphosphine benzoic acid and other chemicals were purchased from Merck and used without any other purifications. Chops of metal gold was purchased from Merck and used to synthesize tetrachloroauric acid by dissolving the gold chop by boiling aqua regia and by the careful evaporation of water till almost to dryness. Me_2_SAuCl was prepared by the reduction of tetrachloroauric acid with an excess of Me_2_S in ethanol. HPLC grade CH_2_Cl_2_ was dried over 3 Å molecular sieves purchased from Supelco before the use. The reactions were led under an inert atmosphere of nitrogen. Brookhart anion was freshly synthesized before its use according to the literature.^49^ L^COOMe^ and L^COOEt^ were synthesized as previously described,^32^ the solvent-mediated preparations of L^COOH^AuCl and (L^COOH^)_2_AuCl were previously reported in the literature by some of the authors.^30^

### Characterizations

Elemental analyses (C, H, N and S) were performed in-house with a Fisons Instruments 1108 CHNS–O Elemental Analyser. Melting points were taken on an SMP3 Stuart Scientific Instrument. IR spectra were recorded from 4000 to 100 cm^−1^ with a PerkinElmer SPECTRUM ONE System FT-IR instrument. IR annotations used: br = broad, m = medium, s = strong, sh = shoulder, vs = very strong, w = weak, and vw = very weak. ^1^H and ^31^P NMR spectra were recorded on an Oxford-400 Varian spectrometer (400.4 MHz for ^1^H and 162.1 MHz for ^31^P) and on a 500 Bruker Ascend (500 MHz for ^1^H, 202.46 for ^31^P). Chemical shifts, in ppm, for ^1^H NMR spectra are relative to internal Me_4_Si. ^31^P NMR chemical shifts were referenced to an 85% H_3_PO_4_ standard. The ^31^P NMR spectroscopic data were accumulated with ^1^H decoupling. NMR annotations used: br = broad, d = doublet, dd = double doublet, t = triplet, m = multiplet, s = singlet. Electrospray mass spectra (ESI-MS) were obtained in positive- or negative-ion mode on a Series 1100 MSD detector HP spectrometer, using an acetonitrile or methanol mobile phase. The compounds were added to reagent-grade acetonitrile to give solutions of approximate concentration of 0.1 mM. These solutions were injected (1 μL) into the spectrometer *via* an HPLC HP 1090 Series II fitted with an auto-sampler. The pump delivered the solutions to the mass spectrometer source at a flow rate of 300 μL min^−1^, and nitrogen was employed both as a drying and nebulizing gas. Capillary voltages were typically 4000 V and 3500 V for the positive- and negative-ion mode, respectively. Confirmation of all major species in this ESI-MS study was aided by a comparison of the observed and predicted isotope distribution patterns, the latter calculated using the IsoPro 3.0 computer program.

### Synthesis of complex L^COOH^AuCl (1)

#### Mechanochemical preparation

Solid L^COOH^ (100 mg; 0.326 mmol) was mixed in a mortar with solid Me_2_SAuCl (96 mg; 0.326 mmol) for 10 minutes with some drops of CH_2_Cl_2_. The reaction was monitored by recording IR spectra and following the shift of the carbonyl band due to the formation of 1, with respect to the starting ligand. The solid was dissolved with CHCl_3_, filtered through celite, and dried at reduced pressure. The solid was then washed with hexane (2 × 2 mL) and diethyl ether (2 × 2 mL) to remove the excess of the starting ligand. The solid once dried appears as a microcrystalline white powder. Crystals suitable for X-ray diffraction were prepared by layering diethyl ether over a methanol solution. Yield 70%. ^1^H NMR (CDCl_3_, *δ*): 8.18 (dd, ^3^*J*_H–H_ = 8 Hz, 2 Hz, 2H), 7.55 (m, 12H). ^31^P NMR (CDCl_3_, *δ*): 33.2 (s, sharp). MIR (cm^−1^): 3072 (w), 3054 (w), 2989 (w), 2949 (w), 2843 (w), 2813 (w), 2662 (w), 2608 (w), 2555 (w), 1682 (vs), 1600 (m), 1562 (m), 1498 (w), 1481 (m), 1435 (m), 1428 (m), 1395 (m), 1360 (w), 1321 (m), 1296 (s), 1186 (m), 1161 (w), 1133 (w), 1103 (s), 999 (m), 950 (br, m), 856 (m), 818 (m), 765 (s), 743 (s), 712 (vs). FIR (cm^−1^): 697 (m), 688 (s), 672 (sh, m), 647 (w), 631 (w), 620 (w), 601 (w), 590 (w), 555 (m), 540 (m), 524 (s), 500 (s), 477 (m), 465 (m), 454 (m), 442 (w), 427 (w), 413 (w), 396 (w), 378 (m), 340 (s), 334 (s), 318 (s), 295 (m), 285 (m), 274 (m), 266 (m), 260 (m), 252 (m), 244 (m), 229 (sh, m), 226 (m), 215 (m), 207 (m). ESI (+) (CH_3_OH, *m*/*z*, relative intensity): 1041 (100) (L^COOH^AuCl + L^COOH^Au)^+^. ESI (−) (CH_3_OH, *m*/*z*, relative intensity): 1075 (100) (L^COO^AuCl + L^COOH^Au)^−^. Elemental analysis calculated for C_19_H_15_ClO_2_PAu (%): C 42.36; H 2.81. Found %: C 42.15; H 2.64.

### Synthesis of complex L^COOMe^AuCl (2)

#### Solvent-mediated synthesis

A solution of L^COOMe^ (160 mg; 0.50 mmol) in CHCl_3_ (6 mL), was added dropwise to a solution of dimethylsulfide gold(i) chloride (147 mg; 0.50 mmol) dissolved in CHCl_3_ (10 mL) at 0 °C. The reaction mixture was stirred for 3 h and then filtered over celite. The product was washed with hexane (3 × 5 mL) and dried at reduced pressure. X-ray quality single crystals were obtained from a methanol/diethylether solution at room temperature. Yield: 92%.

#### Mechanochemical synthesis

Solid L^COOMe^ (60 mg; 0.187 mmol) and dimethylsulfide gold(i) chloride (55 mg; 0.187 mmol), were ground in a mortar for 10 minutes with some drops of dichloromethane. The waxy solid was dissolved with dichloromethane (3 mL), filtered with a PTFE filter (20 μm), and dried at reduced pressure. The solid was washed with hexane (2 × 2 mL), and dried. Yield: 85%. ^1^H NMR (CDCl_3_, *δ*): 8.12 (dd, ^3^*J*_H–H_ = 8 Hz, 2 Hz, 2H), 7.63–7.50 (m, 12H), 3.97 (s, 3H). ^31^P NMR (CDCl_3_, *δ*): 33.08 (s, sharp). MIR (cm^−1^): 3086 (w), 3060 (w), 3035 (w), 3008 (w), 2991 (w), 2950 (w), 2840 (w), 1719 (s), 1619 (w), 1599 (w), 1589 (w), 1562 (w), 1484 (m), 1434 (m), 1392 (m), 1361 (w), 1341 (w), 1317 (m), 1308 (m), 1282 (s), 1273 (s), 1242 (m, sh), 1192 (m), 1177 (m), 1163 (m, sh), 1119 (m), 1097 (s), 1027 (m, sh), 1018 (m), 996 (m), 977 (w), 962 (m), 931 (w), 856 (m), 829 (m), 762 (m, sh), 751 (s), 728 (m), 711 (m). FIR (cm^−1^): 692 (s), 651 (w), 645 (w), 632 (m), 618 (w), 585 (w), 559 (s), 525 (m), 511 (s), 484 (m), 472 (m, sh), 442 (m), 403 (w), 396 (w), 374 (m), 364 (w, sh), 346 (m, sh), 332 (s), 326 (sh, s), 300 (m), 287 (w), 288 (w), 275 (w), 268 (w), 257 (w), 248 (w), 224 (m), 201 (m), 190 (m), 177 (m), 169 (m), 156 (m), 144 (w), 137 (w), 128 (w), 119 (m), 114 (m), 107 (m). ESI (+) (CH_3_OH, *m*/*z*, relative intensity): 1127 (50), 1069 (100), 837 (40) [(L^COOMe^)_2_Au]^+^. Elemental analysis calculated for C_20_H_17_AuClO_2_P (%): C 43.46, H 3.10: found (%): C 43.86, H 3.10.

### Synthesis of complex L^COOEt^AuCl (3)

Solid L^COOEt^ (94 mg; 0.281 mmol) was dissolved in CHCl_3_ (4 mL) and it was added dropwise at 0 °C to dimethylsulfide gold(i) chloride (82 mg; 0.281 mmol) dissolved in 5 mL of CHCl_3_. The solution was stirred for 4 h at room temperature, and then it was dried at reduced pressure. The waxy solid was washed with hexane (3 × 3 mL) and dried at reduced pressure. Yield: 94%. ^1^H NMR (CDCl_3_, *δ*): 8.13 (dd, ^3^*J*_H–H_ = 8 Hz, 2 Hz, 2H), 7.63–7.49 (m, 12H), 4.43 (q, ^3^*J*_H–H_ = 7 Hz, 2H), 1.42 (t, ^3^*J*_H–H_ = 7 Hz, 3H). ^31^P NMR (CDCl_3_, *δ*): 33.02 (s, sharp). MIR (cm^−1^): 3072 (w), 3054 (w), 2979 (w), 2958 (w), 2928 (w), 2904 (w), 2869 (w), 2855 (w), 1715 (s), 1600 (w), 1587 (sh, w), 1575 (w), 1481 (w), 1464 (sh, w), 1436 (m), 1394 (m), 1366 (w), 1335 (w), 1311 (w), 1271 (s), 1183 (m), 1172 (sh, w), 1162 (sh, w), 1122 (sh, m), 1093 (s), 1018 (m), 998 (w), 971 (sh, w), 923 (w), 873 (sh, w), 853 (m), 788 (w), 761 (sh, m), 749 (m), 725 (m), 712 (m). FIR (cm^−1^): 690 (m), 667 (sh, w), 632 (m), 618 (sh, w), 554 (m), 525 (m), 511 (m), 494 (m), 475 (w), 456 (w), 415 (m), 388 (m), 376 (w), 369 (w), 357 (w), 332 (s), 327 (s), 301 (w), 283 (m), 275 (m), 261 (w), 252 (w), 245 (w), 234 (w), 218 (w), 208 (w), 192 (w), 183 (w), 161 (w), 137 (s), 126 (s), 119 (m), 114 (sh, m). ESI (+) (CH_3_OH, *m*/*z*, relative intensity): 1097 (50), 865 (80) [(L^COOEt^)_2_Au]^+^, 525 (35) [(L^COO^ + Na)Au]^+^. ESI (−) (CH_3_OH, *m*/*z*, relative intensity): 267 (100). Elemental analysis calculated for C_21_H_19_AuClO_2_P (%): C 44.50 H 3.38: found (%): C 44.08, H 3.42.

### Synthesis of compound [bis(4-diphenylphosphanyl-benzoate)gold(i) chloride], (L^COOH^)_2_AuCl (4)

Complex 4 was synthesized according to three different procedures. Method A is slightly modified with respect to the procedure already reported by Galassi *et al.*^30^

**Method A**: a solution of L^COOH^ (208 mg; 0.68 mmol) in CHCl_3_ (10 mL) was added dropwise to a solution of Me_2_SAuCl (100 mg, 0.34 mmol) in CHCl_3_ (10 mL) at 0 °C. The mixture was stirred at r. t. for 1.5 hours in the darkness. The mixture was filtered off and washed with CHCl_3_ (3 mL × 3) and dried. The white product was crystallized by dissolving in the minimum amount of CH_3_OH and layering diethyl ether at 4 °C. Yield 98%, m. p. 277 °C. **Method B**: a solution of L^COOH^ (104 mg; 0.34 mmol) in CHCl_3_ (5 mL) was added dropwise to a solution of Me_2_SAuCl (100 mg, 0.34 mmol) in CHCl_3_ (10 mL) at 0 °C. The suspension was left to stir for 1.5 hours and then centrifuged. The raw solid was resuspended in CHCl_3_ (5 mL) and a solution of 4-(diphenylphosphane)benzoic acid (104 mg; 0.34 mmol) in CHCl_3_ (5 mL) was added. The suspension was left to stir for an additional 2 hours. The solid was isolated by filtration and dried. Yield 82%. **Method C**: solid L^COOH^ (20.8 mg; 0.0679 mmol) was mixed in a mortar with solid Me_2_SAuCl (10 mg; 0.0339 mmol) for 30 minutes. The reaction was monitored by recording IR spectra and following the rising of the carbonyl band due to the bis-phosphane Au(i) compounds and the decrease of the carbonyl band of the starting ligand. The solid was recovered and washed with diethyl ether to remove the excess of the starting ligand. The solid once dried appears as a microcrystalline colorless powder. Yield 84%. The microcrystalline solids obtained by the different methods displayed overlapping analytical and spectroscopic characterizations, except for the presence of different solvents used in the procedure. Below, the data for the sample obtained by method A. ^1^H NMR (CD_3_OD, *δ*): 8.09 (d, ^3^*J*_H–H_ = 7.7 Hz, 4H), 7.63 (m, 4H), 7.54 (m, 20H). ^31^P NMR (CD_3_OD, *δ*): 43.57 (s). MIR (cm^−1^): 3749 (w), 3419.4 (w), 3032.8 (m), 2918 (m), 2581.3 (w), 1979.4 (w), 1926.3 (w), 1717 (vs), 1599.2 (m), 1563.2 (m), 1482.6 (m), 1435.7 (s), 1376.7 (s), 1331.5 (s), 1309 (m), 1211.4 (s), 1174.1 (s), 1118.6 (m), 1101.5 (s), 1018.2 (m), 998.3 (m), 850 (m), 799.8 (m), 751 (s), 744.2 (s), 712.2 (m), 709 (s). FIR (cm^−1^): 692 (m), 680 (m), 661 (sh, w), 643 (w), 633 (w), 625 (w), 617 (w), 598 (w), 585 (w), 567 (w), 559 (w), 537 (m), 517 (sh, m), 497 (s), 479 (sh, m), 455 (m), 438 (w), 415 (w), 407 (w), 391 (w), 376 (w), 368 (w), 348 (w), 337 (w), 330 (w), 324 (w), 314 (w), 297 (m), 277 (m), 257 (m), 243 (sh, w), 230 (w), 218 (w), 205 (w), 198 (w), 186 (w), 172 (m), 164 (w), 140 (m), 126 (m), 116 (m), 106 (s). ESI (−) (CH_3_OH) *m*/*z*, %: 537.0 (88) [L^COO^AuCl]^−^, 1074.9 (100) [L^COO^AuCl + L^COOH^AuCl]^−^. ESI (+) (CH_3_OH) *m*/*z* %: 809 (100) [(L^COOH^)_2_Au]^+^. Elemental analysis calculated for C_38_H_30_ClO_4_P_2_Au (%): C 54.46; H 3.87. Found (%): C 54.11; H 3.95.

### Synthesis of compound [bis(4-diphenylphosphanyl-benzoate)gold(i)][trifluoromethanesulfonate], (L^COOMe^)_2_AuOTf (5)

Solid L^COOMe^ (100 mg; 0.312 mmol) was added to a solution of dimethylsulfide gold(i) chloride (46 mg; 0.156 mmol) in dry CH_2_Cl_2_ (3 mL), at 0 °C; the mixture was stirred for 1 h at room temperature. After 1 hour, 1 eq. of L^COOMe^ (100 mg; 0.312 mmol) was added at 0 °C, and the solution was stirred for 1 h at room temperature. The solid silver trifluoromethanesulfonate (40 mg; 0.156 mmol), was added to the mixture, and the suspension, after 1 h of stirring at room temperature in the darkness, was filtered over a celite bed and evaporated at reduced pressure. The waxy solid was washed with hexane (2 × 2 mL), and diethyl ether (3 × 2 mL), and the white precipitate was dried. Yield 76%. ^1^H NMR (CDCl_3_, *δ*): 8.19 (d, ^3^*J*_H–H_ = 8.2 Hz, 4H), 7.69–7.57 (m, 24H), 3.98 (s, 6H). ^31^P NMR (CDCl_3_, *δ*): 44.16 (slightly broad s). MIR (cm^−1^): 3086 (w), 3057 (w), 3024 (w), 3011 (w), 2995 (w), 2953 (w), 2927 (w), 2875 (w), 2846 (w), 1718 (s), 1600 (w), 1587 (w), 1575 (w), 1562 (sh, w), 1482 (w), 1455 (w), 1436 (m), 1395 (m), 1361 (sh, w), 1332 (sh, w), 1313 (sh, w), 1264 (vs), 1223 (sh, s), 1187 (m), 1147 (s), 1119 (sh, w), 1094 (s), 1029 (s), 1017 (sh, m), 998 (m), 961 (sh, m), 924 (w), 854 (m), 829 (w), 761 (sh, m), 750 (s), 727 (s), 714 (m). FIR (cm^−1^): 691 (s), 671 (sh, w), 618 (sh, m), 572 (m), 555 (w), 541 (m), 515 (s), 485 (sh, m), 454 (w), 439 (w), 404 (w), 395 (w), 365 (m), 342 (m), 297 (w), 290 (w), 284 (w), 272 (w), 262 (w), 247 (w), 202 (m), 194 (m), 185 (m), 175 (m), 162 (sh, w), 155 (m), 139 (m), 125 (m), 120 (s), 112 (m) 108 (sh, w). ESI (−) (CH_3_OH) *m*/*z*, %: 149 (100) [CF_3_O_3_S^−^ (triflate)]. ESI (+) (CH_3_OH) *m*/*z* %: 837 (100) [(L^COOMe^)_2_Au]^+^. Elemental analysis calculated for C_41_H_34_AuF_3_O_7_P_2_S + ½CH_2_Cl_2_ (%): C 48.43; H 3.43, S 3.12. Found (%): C 48.80; H 3.19, S 2.86.

### Synthesis of compound [bis(4-diphenylphosphanyl-methylbenzoate)-gold(i)][hexafluorophosphate], (L^COOMe^)_2_AuPF_6_, (6)

A solution of L^COOMe^ (50 mg; 0.156 mmol) in anhydrous CH_2_Cl_2_ (3 mL) was added dropwise in a solution of Me_2_SAuCl (46 mg, 0.156 mmol) in anhydrous CH_2_Cl_2_ (2 mL) at 0 °C. The mixture was stirred at r. t. for 3 hours in the darkness. A second amount of L^COOMe^ (50 mg; 0.156 mmol) in anhydrous CH_2_Cl_2_ (3 mL) was added and stirred for 10 minutes. Afterward, solid AgPF_6_ (40 mg, 0.156 mmol) was added. The suspension was stirred in the darkness for 1.5 hours, filtered upon a celite bed, and evaporated to dryness. The raw solid was washed with toluene (3 × 2 mL) and furtherly with hexane (2 × 2 mL) and dried at reduced pressure. Yield 73%. ^1^H NMR (CDCl_3_, *δ*): 8.17 (d, ^3^*J*_H–H_ = *c* Hz, 2H), 7.66 (m, 2H), 7.55 (m, 24H), 3.95 (s, 6H). ^31^P NMR (CDCl_3_, *δ*): 36.65 (s), −18.38 (t, ^1^*J*_P–F_ = 978 Hz). MIR (cm^−1^): 3057 (w), 3026 (w), 3010 (w), 2995 (w), 2954 (w), 2846 (w), 1719 (vs), 1600 (w), 1589 (sh, w), 1563 (w), 1484 (w), 1436 (s), 1395 (m), 1336 (sh, w), 1313 (sh, w), 1283 (vs), 1187 (m), 1163 (sh, w), 1120 (sh, m), 1100 (s), 1017 (m), 999 (m), 964 (w), 926 (w), 831 (vs), 761 (s), 748 (s), 727 (s) 715 (w). FIR (cm^−1^): 690 (vs), 652 (w), 632 (w), 618 (w), 556 (s), 541 (m), 515 (s), 484 (m, sh), 451 (m), 432 (m), 396 (m), 366 (m), 340 (m), 321 (w, sh) 302 (m), 292 (w), 283 (w), 271 (m), 261 (m), 252 (m), 243 (w), 231 (w), 213 (w), 204 (m), 195 (m), 181 (m), 172 (m), 162 (w), 150 (m), 140 (m), 129 (m), 118 (m), 108 (w). ESI (+) (CH_3_OH, *m*/*z*, relative intensity): 837 (100) [(L^COOMe^)_2_Au]^+^. ESI (−) (CH_3_OH, *m*/*z*, relative intensity): 145 (100) [PF_6_]^−^. Elemental analysis calculated for C_40_H_34_AuF_6_O_4_P_2_ (%): C 48.89, H 3.49. Found: C 49.50, H 3.70.

### Synthesis of compound [bis(4-diphenylphosphanyl-methylbenzoate)gold(i)][tetrakis(3,5-bis(trifluoromethyl)phenyl)borate], (L^COOMe^)_2_AuBAr_4_, (7)

L^COOMe^ (16.5 mg, 0.092 mmol) was dissolved in dry CH_2_Cl_2_ (2 mL) and added to a solution of dimethylsulfide gold(i) chloride (27 mg; 0.092 mmol) in CH_2_Cl_2_ (2 mL) at 0 °C. After 3 hours of magnetic stirring at r. t. another equivalent of L^COOMe^ (16.5 mg, 0.092 mmol) was added at 0 °C. The reaction was stirred for 10 minutes at r. t. and transferred into a round bottom flask, containing freshly prepared solid AgBAr_4_ (89.4 mg: 0.092 mmol). Upon dissolution of the silver salt, a white precipitate (AgCl) was formed. The reaction was stirred for 2 h at r. t. in the darkness. The suspension was then filtered over a celite bed (3 cm) and dried at reduced pressure. The crude product was washed with hexane (3 × 3 mL) and dried at reduced pressure. X-ray quality crystals were grown by slow diffusion of Et_2_O in a methanol solution at 5 °C. Yield 45%. ^1^H-NMR (CDCl_3_): 8.18 (d, 4H, ^3^*J*_H–H_ = 8 Hz), 7.69 (s, br, 12H), 7.60–7.47 (m, 24H), 3.94 (s, 6H). ^31^P-NMR (CDCl_3_): 46.10 (s). MIR (cm^−1^): 3075 (w), 3031 (w), 3004 (w), 2957 (w), 1733 (s), 1607 (w), 1563 (w), 1486 (w), 1463 (w), 1439 (m), 1397 (w), 1353 (s), 1314 (sh, w), 1272 (vs), 1162 (sh, m), 1117 (s), 1104 (s), 1096 (s), 1028 (w), 1018 (m), 1000 (m), 970 (w), 940 (w), 930 (w), 895 (m), 857 (w), 839 (m), 829 (w), 762 (m), 746 (m), 728 (s), 713 (s). FIR (cm^−1^): 692 (s), 682 (s), 671 (s), 651 (w), 632 (w), 618 (w), 581 (w), 551 (w), 540 (m), 531 (m), 520 (s), 508 (m), 490 (m), 468 (w), 449 (m), 404 (w), 395 (w), 381 (w), 364 (m), 348 (m), 321 (w), 294 (sh, w), 285 (w), 272 (w), 256 (w), 238 (w), 214 (w), 202 (m), 195 (w), 184 (w), 174 (w), 163 (w), 159 (w), 150 (w), 139 (w), 129 (w), 117 (m), 105 (s). ESI (+) (CH_3_OH, *m*/*z*): 837 (100) [(L^COOMe^)_2_Au]^+^. ESI (−) (CH_3_OH, *m*/*z*): 863 (100) [BAr_4_]^−^. Elemental analysis calculated for C_72_H_46_AuBF_24_O_4_P_2_ (%): C 50.84, H 2.65. Found: C 50.55 H 2.83.

### Synthesis of compound [bis(4-diphenylphosphanyl-ethylbenzoate)gold(i)][hexafluorophosphate], (L^COOEt^)_2_AuPF_6_, (8)

A solution of L^COOEt^ (45 mg; 0.134 mmol) in CH_2_Cl_2_ (3 mL) was added dropwise in a solution of dimethylsulfide gold(i) chloride (39.6 mg, 0.134 mmol) in anhydrous CH_2_Cl_2_ (2 mL) at 0 °C. The mixture was stirred at r. t. for 3 hours in the darkness. A second equivalent of L^COOEt^L (45 mg; 0.134 mmol) dissolved in CH_2_Cl_2_ (3 mL) was added and the solution was left to stir for 10 minutes. Afterward, solid AgPF_6_ (34 mg, 0.134 mmol) was added. The suspension was stirred in the darkness for 1.5 hours, filtered upon a celite bed, and evaporated to dryness. The raw solid (110 mg) was washed with hexane (4 × 3 mL) and dried at reduced pressure. Yield 81%. ^1^H NMR (CDCl_3_, *δ*): 8.22 (d, ^3^*J*_H–H_ = 5 Hz, 4H), 7.68–7.57 (m, 24H), 4.43 (q, ^3^*J*_H–H_ = 8 Hz, 4H), 1.43 (t, ^3^*J*_H–H_ = 8 Hz, 6H). ^31^P NMR (CDCl_3_, *δ*): 44.82 (s), −144.31 (spt, ^1^*J*_P–F_ = 713 Hz, PF_6_^−^). MIR (cm^−1^): 3077 (w), 3058 (w), 2983 (w), 2958 (w), 2929 (w), 2906 (w), 2872 (w), 2855 (w), 1714 (m), 1600 (w), 1597 (w), 1587 (sh, w), 1564 (w), 1483 (w), 1465 (w), 1438 (m), 1395 (m), 1336 (m), 1313 (w, sh), 1273 (s), 1185 (m), 1163 (w, sh), 1125 (m, sh), 1094 (s), 1018 (m), 999 (m), 928 (w), 877 (m, sh), 832 (vs), 760 (m), 748 (m), 726 (m) 713 (m). FIR (cm^−1^): 690 (s), 651 (w), 632 (w), 617 (w), 556 (s), 542 (m), 514 (s), 493 (m, sh), 455 (m), 414 (m), 387 (m), 351 (m), 322 (m), 296 (w), 285 (w), 271 (w), 252 (w), 245 (w), 235 (w), 216 (w), 202 (w), 195 (m), 187 (m), 183 (m), 172 (m), 159 (m), 140 (m), 133 (w, sh), 125 (w), 116 (m), 107 (m). ESI (+) (CH_3_OH, *m*/*z*, relative intensity): 865 (100) [(L^COOEt^)_2_Au]^+^. ESI (−) (CH_3_OH, *m*/*z*, relative intensity): 145 (100) [PF_6_]^−^. Elemental analysis calculated for C_42_H_38_AuF_6_O_4_P_3_ (%): C 49.91, H 3.79. Found: C 50.38, H 3.83.

### Synthesis of compound [bis(4-diphenylphosphanyl-ethylbenzoate)gold(i)][tetrakis(3,5-bis(trifluoromethyl)phenyl)borate], (L^COOEt^)_2_AuBAr_4_, (9)

A solution of L^COOEt^ (40 mg; 0.197 mmol) in CH_2_Cl_2_ (3 mL) was added dropwise into a solution of Me_2_SAuCl (17.6 mg, 0.098 mmol) in anhydrous CH_2_Cl_2_ (2 mL) at 0 °C. The mixture was stirred at r. t. for 3 hours in the darkness. Afterward, the solution was transferred into a round bottom flask, containing solid freshly prepared AgBAr_4_ (34 mg, 0.0.98 mmol). The suspension was stirred in the darkness for 1.5 hours, filtered upon a celite bed, and evaporated to dryness. An oily product was obtained and washed with hexane (3 × 3 mL) and dried at reduced pressure. Yield 42%. ^1^H NMR (CDCl_3_, *δ*): 8.23 (d, ^3^*J*_H–H_ = 8 Hz, 4H), 7.72 (s, br, 8H), 7.76–7.49 (m, 28H), 4.44 (q, ^3^*J*_H–H_ = 8 Hz, 4H), 1.41 (t, ^3^*J*_H–H_ = 8 Hz, 6H). ^31^P NMR (CDCl_3_, *δ*): 44.95 (s). MIR (cm^−1^): 3070 (w), 3022 (w), 2987 (w), 2967 (w), 2935 (w), 2910 (w), 1722 (m), 1716 (m), 1611 (w), 1565 (w), 1485 (w), 1464 (w), 1439 (m), 1396 (w), 1354 (s), 1312 (w, sh), 1272 (vs), 1158 (sh, s), 1117 (vs), 1090 (sh, s), 1019 (m), 999 (m), 929 (w), 886 (m), 838 (w), 762 (m), 745 (m), 726 (m), 712 (s). FIR (cm^−1^): 692 (s), 681 (s), 670 (s), 651 (w), 633 (w), 618 (w), 584 (w), 566 (w), 542 (m), 516 (s), 493 (m), 469 (m), 457 (m), 449 (m), 403 (w), 394 (m), 385 (m), 366 (m), 353 (m), 347 (m), 333 (m), 321 (w), 307 (w), 299 (w), 285 (w), 272 (w), 262 (w), 252 (w), 245 (w), 231 (w), 215 (w), 203 (m), 188 (m); 174 (w), 162 (w), 151 (s), 140 (m), 124 (w), 115 (m), 108 (m). ESI (+) (CH_3_OH, *m*/*z*, relative intensity): 865 (98) [(L^COOEt^)_2_Au]^+^, 425 (100). ESI (−) (CH_3_OH, *m*/*z*, relative intensity): 863 (100) [BAr_4_]^−^, 471 (35). Elemental analysis for calculated for C_74_H_50_AuBF_24_O_4_P_2_ (%), C 51.41, H 2.92: found (%): C 51.67, H 2.33.

### Crystallography

Crystals of 2 and 4 were mounted in inert oil on a MiteGen MicroMount and transferred to a Bruker APEX-II CCD diffractometer, equipped with an Oxford Instruments low-temperature controller system (Mo Kα = 0.71073 Å, graphite monochromator). A well-formed single crystal of compound 7 was mounted on a glass fiber and transferred to an APEX II Bruker CCD diffractometer (Mo Kα = 0.71073 Å, graphite monochromator). The APEX 3 program package^50^ was used to obtain the unit-cell geometrical parameters and for the data collection (30 s per frame scan time for a sphere of diffraction data). The raw frame data were processed using SAINT and SADABS^50^ to obtain the data file of the reflections. The structure of 7 was solved using SHELXT (Intrinsic Phasing method in the APEX 3 program).^51^ The refinement of the structure (based on *F*^2^ by full-matrix least-squares techniques) was carried out using the SHELXTL-2014/7 program^52^ in the WinGX suite v.2014.1.^53^ The hydrogen atoms were introduced in the refinement in defined geometry and refined ''riding'' on the corresponding carbon atoms. Data were collected in ω- and φ-scan modes. Absorption effects were treated by semiempirical corrections based on multiple scans. Structures 1, 2, and 4 were solved with the XT structure solution program using intrinsic phasing and refined on F02 with SHELXL-97.^54^ All non-hydrogen atoms were treated anisotropically, and all hydrogen atoms were included as riding bodies.

### Photophysical properties

Diffuse reflectance UV-vis spectra of pressed powder samples diluted with KBr were recorded on a Shimadzu (UV-3600 spectrophotometer with a Harrick Praying Mantis accessory) and recalculated following the Kubelka–Munk function. Excitation and emission spectra in the solid state, as well as lifetime measurements, were recorded with an Edinburgh FLS 1000 fluorescence spectrometer. Quantum yields were measured in the solid state using a Hamamatsu Quantaurus-QY C11347-11 integrating sphere with different excitation wavelengths.

### Computational details

Model systems 4a and 4b were built from the X-ray diffraction data for complex 4, and were fully optimized at the DFT level of theory using the PBE functional^55^ as implemented in TURBOMOLE 6.4.^56^ TD-DFT calculations were performed to compute the lowest singlet-triplet excitation for the model systems.^57–61^ In all calculations, the Karlsruhe split-valence quality basis sets SV(P) were used for C, O, and H,^62^ whereas def2-TZVP basis sets were used for Au, P, and Cl.^63^

## Conclusions

In this work, the mechanochemical preparation of some neutral mono- or bis-phosphane gold(i) derivatives was performed. The results were discussed and compared with those obtained by classical methods, involving the use of organic solvents. By using esters- or the carboxyl-substituted triarylphosphane as ligands, different results were obtained. The outcomes converge to indicate the 4-diphenylphosphanyl-benzoic acid, L^COOH^, as the most effective ligand in the solvent-free preparations both in terms of yield and reproducibility. The solids obtained by both methods are visible light emissive materials upon UV wavelength excitation. The X-ray crystal structure determinations highlighted three different structural motifs at the gold center: linear neutral (1 and 2) or linear cationic (7) and T-shaped neutral (4) structures. As expected for tri-coordinate gold(i) phosphane compounds,^12,13^ the T-shaped gold(i) bis-phosphane compound with L^COOH^, compound 4, is a green emitter after excitation in the UV range of wavelengths; however, the analyses of the spectra and the corroboration of them by DFT calculations suggest a structural model consisting of an oligomer of 4, made of three independent units of 4 aggregated by hydrogen bonding; the optimal match between the theoretical aggregation model and the experimental photophysical data, promotes the supramolecular structural architecture as the key factor for the intense green emission of these compounds. Remarkably, d^10^–d^10^ metallophilic bonding, often found for gold(i) compounds similar to those herein discussed, are completely absent in the discussed structures.

Additionally, this work provides some hints on a new catalytic activity of gold(i) phosphane compounds. In this regard, mononuclear cationic bis-phosphane gold(i) compounds have been prepared with different anions, to evidence an unprecedented catalytic role of the bis-phosphane gold(i) compound containing the L^COOMe^, 4-diphenylphosphanyl-methoxy benzoate ester, compound 6, in the hydrolysis of the PF_6_ anion, earlier attained only with square planar palladium complexes.^41^

## Author contributions

RG and LL conceived the study and designed the experiments. LL performed the preparations and the characterizations. NS prepared and growed crystals for complex 4. Photophysical and computational studies were led by JM-d-L, MR-C and MM. X-ray data were acquired and solved by MR-C, excluding complex 7 which was made by CG. The original draft was written and revised by RG, LL, and JLDL. Supervision was led by RG. All authors discussed the results and commented on the manuscript.

## Conflicts of interest

There are no conflicts to declare.

## Supplementary Material

RA-013-D3RA03681B-s001

RA-013-D3RA03681B-s002
