# Supplementary material for: Mechanochemical preparation of strongly emissive monosubstituted triarylphosphane gold(i) compounds activated by hydrogen bonding driven aggregations
Source: RSC Adv. 2023 Aug 24;13(36):25425–36. doi: 10.1039/d3ra03681b (PMC10448354; doi:10.1039/d3ra03681b)
Supplement: RA-013-D3RA03681B-s001 [file RA-013-D3RA03681B-s001.pdf]

Electronic Supplementary Material (ESI) for RSC Advances.
This journal is © The Royal Society of Chemistry 2023

Mechanochemical preparation of strongly emissive monosubstituted triarylphosphane gold(I) compounds activated by hydrogen bonding driven aggregations

*Lorenzo Luciani,[a] Nicola Sargentoni,[a] Claudia Graiff,[b] Miguel Monge,[c] María Rodríguez-Castillo,[c] Josè Lopez de Luzuriaga,[c] Rossana Galassi, [a]**

University of Camerino, School of Science and Technology, Chemistry Division, c/o ChIP, Via Madonna delle Carceri, 10, Camerino, I-62032, Italy
[b] Department of Chemistry, Life Sciences and Environmental Sustainability, Product Development.Università degli Studi di Parma, Parco Area delle Scienze 17/A, Parma, I-43124, Italy
[c] Universidad de La Rioja, Departamento de Química, Área de Química Inorgánica,Centro de Investigación en Síntesis Química Complejo Científico-Tecnológico Madre de Dios, 51, 26004 Logroño (La Rioja), España-Spain.

Supporting Information

Table 1S. The table reports the list of a) the C=O stretching mode wavenumbers recorded for the mono- and bis-phosphane complexes, b) $\Delta_{C=O}$ values ($\Delta_{C=O}$ = difference of wavenumbers between the complex and the starting ligands), c) ^{31}P NMR resonances of the mono- and bis-phosphane complexes in $CDCl_3$, and d) Δ_{ppm} values (Δ_{ppm} = chemical shifts' change upon coordination of the ligand to the metal).

Compounds	C=O stretching mode, cm^{-1}	$\Delta_{C=O}$ cm^{-1}	^{31}P NMR [a](Full Width at Half Maximum)	Δ_{ppm} ^{31}P NMR
**L**			4.70 [a]	
**L^{COOH}**	1680	-	-4.68, -5.33[b]	-
**L^{COOMe}**	1719	-	-5.01	-
**L^{COOEt}**	1714	-	-5.16	-
L^{COOH}AuCl (**1**)	1682	+2	33.22	+37.90
L^{COOMe}AuCl (**2**)	1719	0	33.08	+39.14
L^{COOEt}AuCl (**3**)	1719	0	33.02	+39.14
(L^{COOH})$_2$AuCl (**4**)*	1717	+37	43.57[a] (109 Hz)	+38.34[c]
(L^{COOMe})$_2$AuOTf (**5**)	1718	-1	44.16 (125 Hz)	+49.18
(L^{COOMe})$_2$AuPF$_6$ (**6**)	1719	0	36.65 (160 Hz)	+41.66[d]
(L^{COOMe})$_2$AuBAr$_4$ (**7**)	1733	+14	46.10 (9 Hz)	+51.11
(L^{COOEt}) $_2$AuPF$_6$ (**8**)	1719	+5	44.82 (10 Hz)	+50.31
(L^{COOEt})$_2$AuBAr$_4$ (**9**)	1722	+8	44.95 (2 Hz)	+50.44

[a] taken from Boris Maryasina and Hendrik Zipse, *Phys. Chem. Chem. Phys.,* 2011, **13**, 5150-5158.

[b] The (L^{COOH})$_2$AuCl (**4**) ^{31}P NMR resonance is reported in CD_3OD at 293 K, due to the low solubility of the complex in $CDCl_3$.

[c] the difference is between the ^{31}P NMR signals in CD_3OD.

[d] The PF_6 ion resulted to be completely hydrolysed in the deuterated solvent to afford the PO_2F_2 anion during the ^{31}P NMR recording.

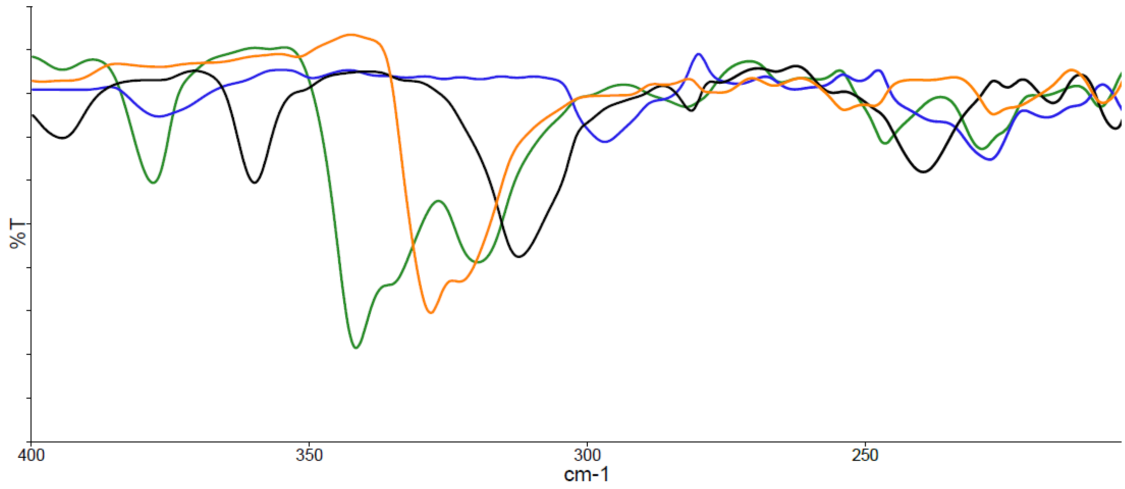

Figure 1S. Overlapped Far-IR spectra in the range 400-200 cm^{-1} for L^{COOH} (black line), L^{COOH}AuCl (**1**) (green line), $(L^{COOH})_2$AuCl (**4**) (blue line). PPh_3AuCl (orange line). The PPh_3PAuCl was added as a reference for the stretching modes of the linear P-Au-Cl bonds.

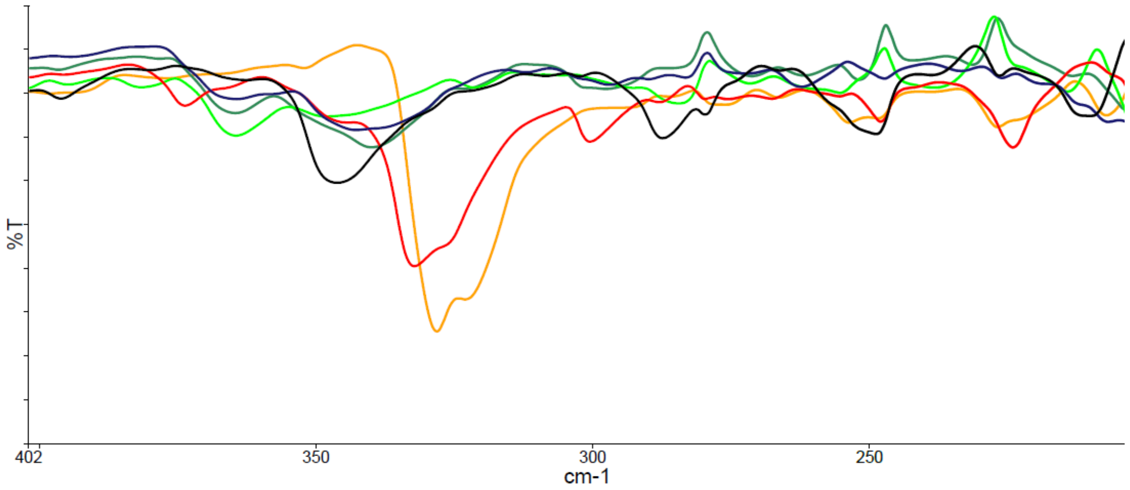

Figure 2S. Overlapped Far-IR spectra in the range 400-200 cm^{-1} for L^{COOMe} (black line) L^{COOMe}AuCl (**2**) (red line), $(L^{COOMe})_2$AuOTf (**5**) (dark blue line) $(L^{COOMe})_2$AuPF$_6$ (**6**) (dark green line), $(L^{COOMe})_2$AuBAr$_4$ (**7**) (light green line). PPh_3AuCl (orange line). The PPh_3PAuCl was added as a reference for the stretching modes of the linear P-Au-Cl bonds.

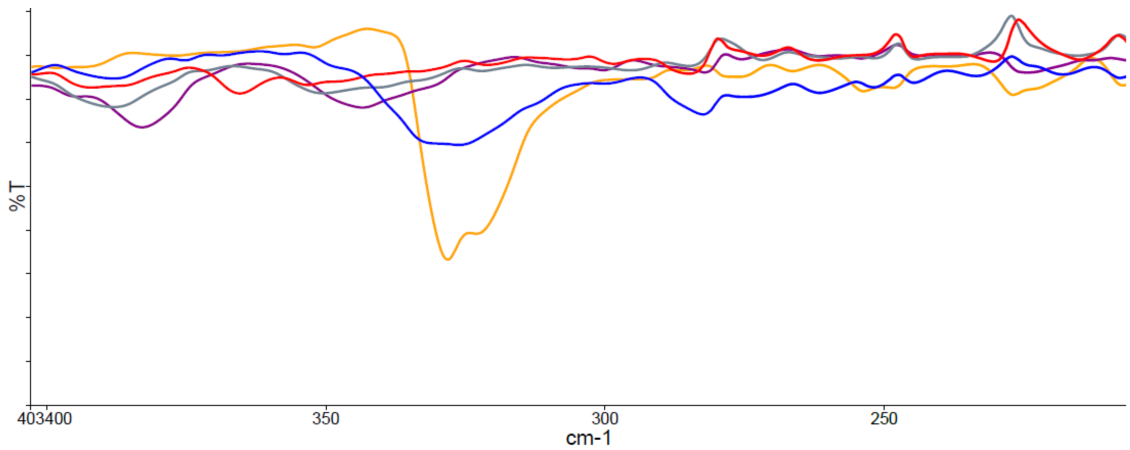

Figure 3S. Overlapped Far-IR spectra in the range 400-200 cm^{-1} for L^{COOEt} (violet line), $L^{COOEt}AuCl$ (**3**) (blue line), $(L^{COOEt})_2AuPF_6$ (**8**) (grey line), $(L^{COOEt})_2AuBAr_4$ (**9**) (red line). PPh_3AuCl (orange line). The PPh_3PAuCl was added as a reference for the stretching modes of the linear P-Au-Cl bonds.

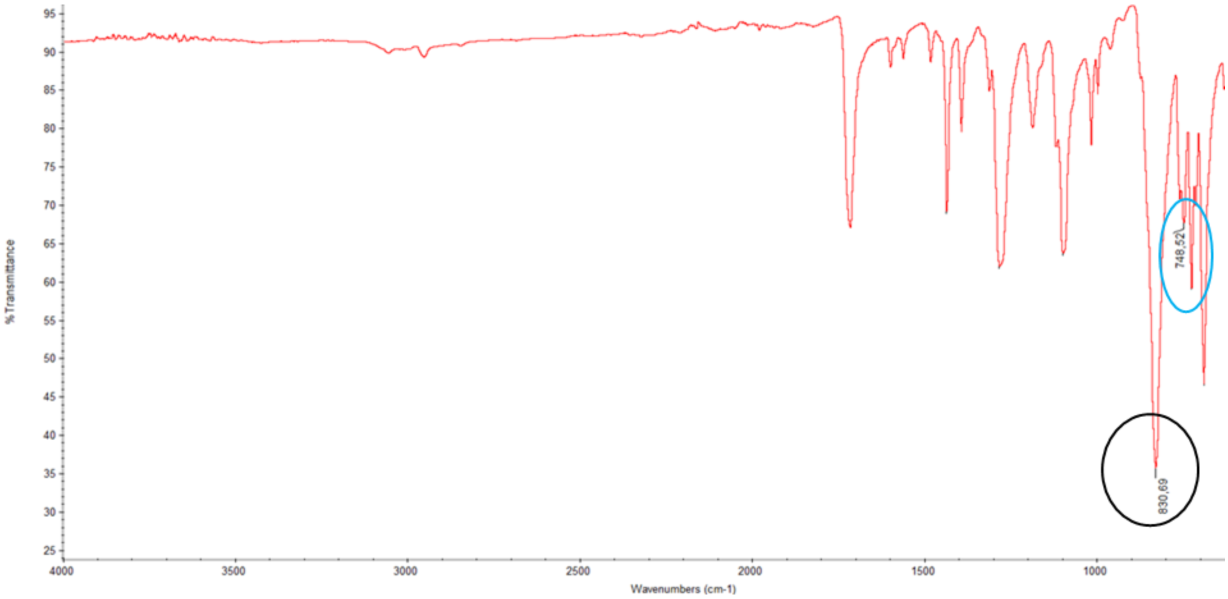

Figure 4S. Mid-IR spectrum of the complex $(L^{COOMe})_2AuPF_6$ (**6**). The asymmetric and the symmetric stretching modes of PF_6^- are highlighted at 830 cm^{-1} (black circle) and 748 cm^{-1} (blue circle), respectively.

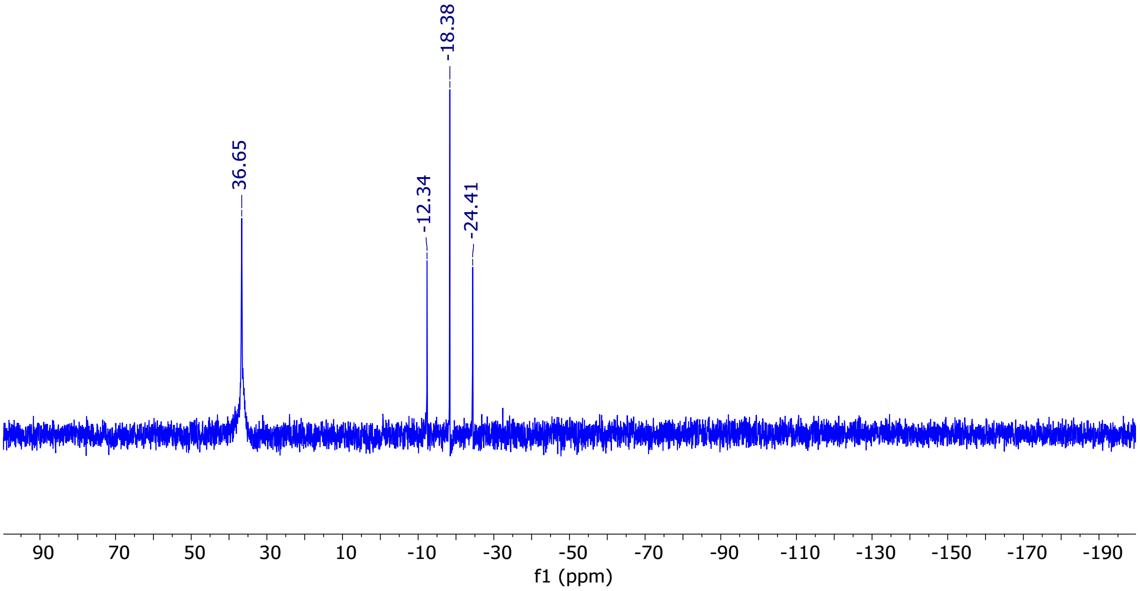

Figure 5S. ^{31}P NMR of complex $(L^{COOMe})_2AuPF_6$ (**6**) in $CDCl_3$ at r. t., showing the complete hydrolysis of the PF_6^- to the PO_2F_2- anion.

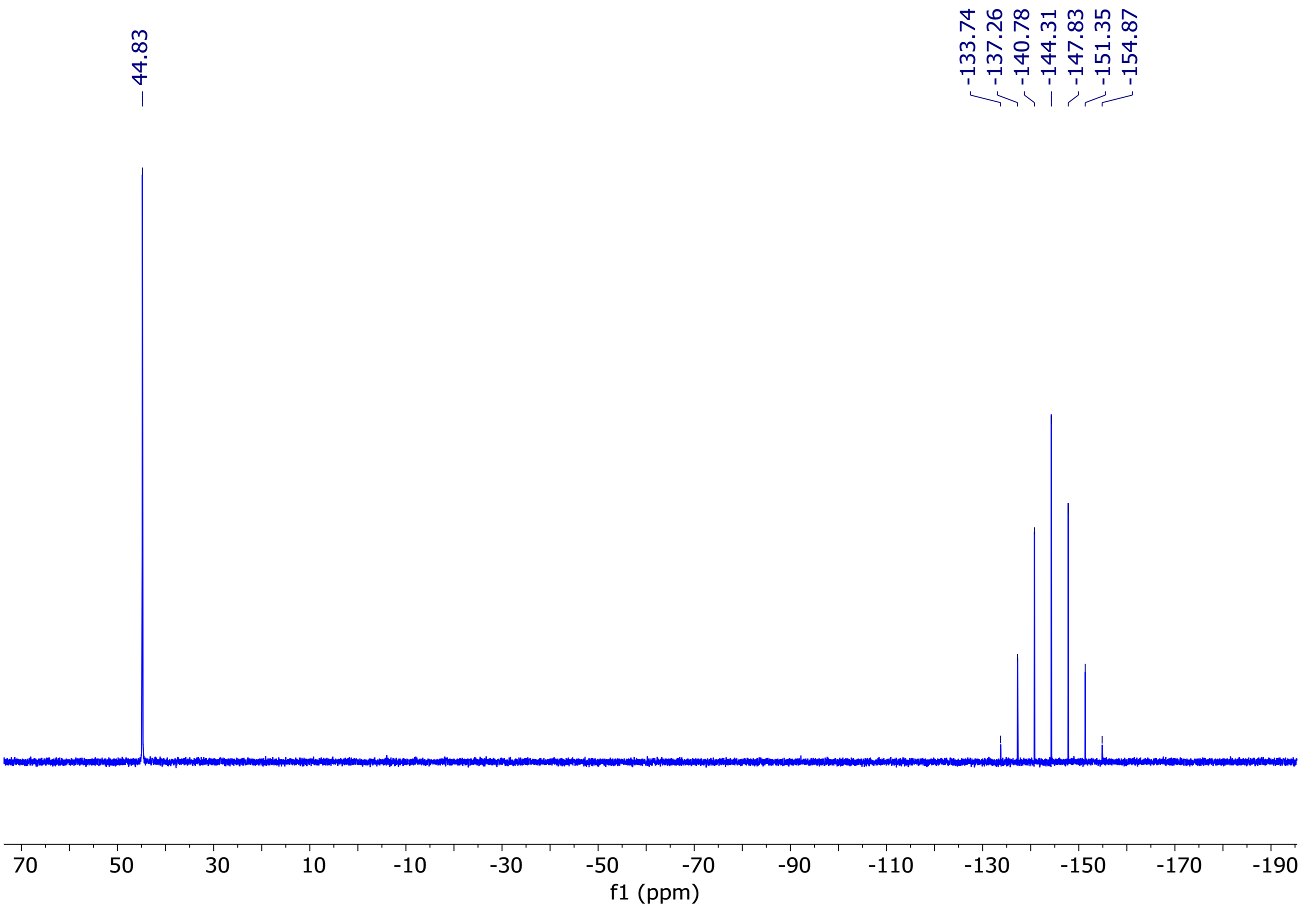

Figure 6S. ^{31}P NMR of complex $(L^{COOEt})_2AuPF_6$ (**8**) in $CDCl_3$ at r. t.

Table 2S. Crystal data for compounds **1**, **2**, **4** and **7**.

**Compounds**	**1**	**2**	**4**	**7**
Formula	$C_{19}H_{15}AuClO_2P$	$C_{20}H_{17}AuClO_2P$	$C_{38}H_{30}AuClO_4P_2,CH_4O$	$C_{72}H_{46}AuBF_{24}O_4P_2$
Space group	P1	P2$_1$/c	C2/c	P2$_1$/c
Crystal system	Triclinic	Monoclinic	Monoclinic	Monoclinic
Cell length (Å)	**a** 12.2536(4) **b** 12.5439(4) **c** 13.9013(5)	**a** 11.7061(5) **b** 11.0279(4) **c** 15.3848(6)	**a** 25.3936(11) **b** 11.0814(4) **c** 15.6192(6)	**a** 24.900(6) **b** 16.943(3) **c** 17.711(5)
Cell Angles (°)	**α** 112.2670(10); **β** 91.5850(10); **γ** 109.1630(10)	**α** 90; **β** 104.7930(10); **γ** 90	**α** 90; **β** 120.8470(10); **γ** 90	**α** 90 **β** 106.237(7) **γ** 90
Cell Volume (Å^3)	1839.36	1920.25	3773.44	7173.9
Units in cell	**Z**: 4 **Z'**: 0	**Z**: 4 **Z'**: 0	**Z**: 4 **Z'**: 0	**Z**: 4 **Z'**: 0
R factor	3.14	3.08	3.88	5.23
Symmetry operations				

Table 3S. Bond length (Å) and angles (°) for compound **1**. Data were compared with those obtained in the literature by F. Mohr, M.C. Jennings, and R. J. Puddephatt, *Angew. Chem. Int. Ed.* 2004, **43**, 969. https://doi.org/10.1002/anie.200353127.

Atom1	Atom2	Length	Atom1	Atom2	Atom3	Angle
Au1	Cl1	2.270(2)	Cl1	Au1	P1	177.72(6)
Au1	P1	2.225(1)	Au1	P1	C1	112.7(2)
P1	C1	1.824(6)	Au1	P1	C7	112.7(2)
P1	C7	1.810(4)	Au1	P1	C13	114.2(2)
P1	C13	1.817(4)	C1	P1	C7	106.9(2)
O1	H1	0.6(1)	C1	P1	C13	105.9(2)
O1	C19	1.269(9)	C7	P1	C13	103.7(2)
O2	C19	1.265(6)	H1	O1	C19	121(10)
C1	C2	1.369(9)	P1	C1	C2	117.4(5)
C1	C6	1.363(8)	P1	C1	C6	123.3(4)
C2	H2	0.93	C2	C1	C6	119.3(6)
C2	C3	1.39(1)	C1	C2	H2	120.5
C3	H3	0.93	C1	C2	C3	119.1(7)
C3	C4	1.35(1)	H2	C2	C3	120.5
C4	H4	0.93	C2	C3	H3	119.6
C4	C5	1.353(9)	C2	C3	C4	120.9(8)
C5	H5	0.93	H3	C3	C4	119.6
C5	C6	1.37(1)	C3	C4	H4	120.1
C6	H6	0.93	C3	C4	C5	119.8(7)
C7	C8	1.369(8)	H4	C4	C5	120.1
C7	C12	1.396(6)	C4	C5	H5	120
C8	H8	0.93	C4	C5	C6	120.1(6)
C8	C9	1.393(8)	H5	C5	C6	119.9
C9	H9	0.93	C1	C6	C5	120.8(6)
C9	C10	1.351(8)	C1	C6	H6	119.6
C10	H10	0.93	C5	C6	H6	119.6
C10	C11	1.36(1)	P1	C7	C8	122.5(4)
C11	H11	0.93	P1	C7	C12	118.7(4)
C11	C12	1.379(8)	C8	C7	C12	118.8(5)
C12	H12	0.93	C7	C8	H8	119.9
C13	C14	1.404(9)	C7	C8	C9	120.3(6)
C13	C18	1.383(6)	H8	C8	C9	119.8
C14	H14	0.929	C8	C9	H9	120
C14	C15	1.368(8)	C8	C9	C10	120.0(6)
C15	H15	0.93	H9	C9	C10	120
C15	C16	1.391(6)	C9	C10	H10	119.7
C16	C17	1.385(8)	C9	C10	C11	120.7(7)
C16	C19	1.473(7)	H10	C10	C11	119.6
C17	H17	0.93	C10	C11	H11	119.8
C17	C18	1.388(8)	C10	C11	C12	120.2(6)
C18	H18	0.93	H11	C11	C12	119.9
Au2	Cl2	2.266(1)	C7	C12	C11	119.9(6)

Au2	P2	2.225(1)	C7	C12	H12	120.1
P2	C20	1.813(6)	C11	C12	H12	120
P2	C26	1.823(5)	P1	C13	C14	117.1(4)
P2	C32	1.822(4)	P1	C13	C18	123.8(4)
O3	H3A	0.73(7)	C14	C13	C18	119.0(4)
O3	C37	1.306(6)	C13	C14	H14	119.7
O4	C37	1.228(7)	C13	C14	C15	120.6(5)
C00P	H00P	0.93	H14	C14	C15	119.7
C00P	C32	1.374(7)	C14	C15	H15	119.8
C00P	C36	1.378(7)	C14	C15	C16	120.3(5)
C20	C21	1.389(6)	H15	C15	C16	119.9
C20	C25	1.375(7)	C15	C16	C17	119.3(5)
C21	H21	0.931	C15	C16	C19	119.9(4)
C21	C22	1.38(1)	C17	C16	C19	120.7(4)
C22	H22	0.93	C16	C17	H17	119.8
C22	C23	1.36(1)	C16	C17	C18	120.5(5)
C23	H23	0.93	H17	C17	C18	119.7
C23	C24	1.377(9)	C13	C18	C17	120.1(5)
C24	H24	0.93	C13	C18	H18	120
C24	C25	1.37(1)	C17	C18	H18	119.9
C25	H25	0.931	O1	C19	O2	122.7(5)
C26	C27	1.375(9)	O1	C19	C16	118.0(5)
C26	C31	1.370(8)	O2	C19	C16	119.3(5)
C27	H27	0.93	Cl2	Au2	P2	178.92(6)
C27	C28	1.391(9)	Au2	P2	C20	113.3(2)
C28	H28	0.93	Au2	P2	C26	112.7(2)
C28	C29	1.347(9)	Au2	P2	C32	112.6(2)
C29	H29	0.93	C20	P2	C26	107.1(2)
C29	C30	1.38(1)	C20	P2	C32	104.6(2)
C30	H30	0.93	C26	P2	C32	105.9(2)
C30	C31	1.38(1)	H3A	O3	C37	108(6)
C31	H31	0.93	H00P	C00P	C32	119.6
C32	C33	1.390(8)	H00P	C00P	C36	119.6
C33	H33	0.93	C32	C00P	C36	120.8(5)
C33	C34	1.369(7)	P2	C20	C21	121.4(4)
C34	H34	0.93	P2	C20	C25	119.5(4)
C34	C35	1.386(7)	C21	C20	C25	119.1(5)
C35	C36	1.394(8)	C20	C21	H21	120.2
C35	C37	1.476(6)	C20	C21	C22	119.7(5)
C36	H36	0.93	H21	C21	C22	120.1
			C21	C22	H22	120.1
			C21	C22	C23	119.7(6)
			H22	C22	C23	120.2
			C22	C23	H23	119.4
			C22	C23	C24	121.2(7)
			H23	C23	C24	119.4
			C23	C24	H24	120.6
			C23	C24	C25	118.8(7)

H24	C24	C25	120.6
C20	C25	C24	121.5(6)
C20	C25	H25	119.3
C24	C25	H25	119.2
P2	C26	C27	121.7(4)
P2	C26	C31	118.7(4)
C27	C26	C31	119.6(5)
C26	C27	H27	120.1
C26	C27	C28	119.7(5)
H27	C27	C28	120.2
C27	C28	H28	119.7
C27	C28	C29	120.7(6)
H28	C28	C29	119.7
C28	C29	H29	120
C28	C29	C30	120.0(7)
H29	C29	C30	119.9
C29	C30	H30	120.2
C29	C30	C31	119.7(7)
H30	C30	C31	120.2
C26	C31	C30	120.3(6)
C26	C31	H31	119.9
C30	C31	H31	119.8
P2	C32	C00P	123.5(4)
P2	C32	C33	117.4(4)
C00P	C32	C33	119.1(5)
C32	C33	H33	119.7
C32	C33	C34	120.7(5)
H33	C33	C34	119.7
C33	C34	H34	119.9
C33	C34	C35	120.2(5)
H34	C34	C35	119.9
C34	C35	C36	119.3(5)
C34	C35	C37	119.3(4)
C36	C35	C37	121.4(4)
C00P	C36	C35	119.8(5)
C00P	C36	H36	120.1
C35	C36	H36	120
O3	C37	O4	122.6(5)
O3	C37	C35	115.7(4)
O4	C37	C35	121.7(4)

Table 4S. Bond length (Å) and angles (°) for compound **2**.

Atom1	Atom2	Length	Atom1	Atom2	Atom3	Angle
Au1	Cl1	2.284(2)	Cl1	Au1	P1	177.35(5)
Au1	P1	2.231(1)	Au1	P1	C1	110.4(2)
P1	C1	1.813(5)	Au1	P1	C7	114.3(2)
P1	C7	1.821(5)	Au1	P1	C13	113.9(2)

P1	C13	1.811(4)	C1	P1	C7	105.3(2)
O1	C19	1.195(6)	C1	P1	C13	106.6(2)
O2	C19	1.316(6)	C7	P1	C13	105.7(2)
O2	C20	1.456(7)	C19	O2	C20	116.4(4)
C1	C2	1.367(7)	P1	C1	C2	123.2(4)
C1	C6	1.379(6)	P1	C1	C6	117.7(4)
C2	H2	0.93	C2	C1	C6	119.0(5)
C2	C3	1.378(8)	C1	C2	H2	119.9
C3	H3	0.931	C1	C2	C3	120.0(6)
C3	C4	1.34(1)	H2	C2	C3	120
C4	H4	0.93	C2	C3	H3	119.1
C4	C5	1.38(1)	C2	C3	C4	121.8(7)
C5	H5	0.93	H3	C3	C4	119.1
C5	C6	1.389(8)	C3	C4	H4	120.4
C6	H6	0.931	C3	C4	C5	119.2(7)
C7	C8	1.365(8)	H4	C4	C5	120.4
C7	C12	1.388(7)	C4	C5	H5	120.3
C8	H8	0.93	C4	C5	C6	119.5(6)
C8	C9	1.39(1)	H5	C5	C6	120.3
C9	H9	0.93	C1	C6	C5	120.5(5)
C9	C10	1.36(1)	C1	C6	H6	119.7
C10	H10	0.93	C5	C6	H6	119.8
C10	C11	1.36(1)	P1	C7	C8	118.6(4)
C11	H11	0.931	P1	C7	C12	122.4(4)
C11	C12	1.374(9)	C8	C7	C12	119.1(5)
C12	H12	0.931	C7	C8	H8	120
C13	C14	1.388(7)	C7	C8	C9	119.9(6)
C13	C18	1.390(7)	H8	C8	C9	120.1
C14	H14	0.93	C8	C9	H9	119.7
C14	C15	1.377(6)	C8	C9	C10	120.7(7)
C15	H15	0.93	H9	C9	C10	119.6
C15	C16	1.383(6)	C9	C10	H10	120.2
C16	C17	1.386(7)	C9	C10	C11	119.6(7)
C16	C19	1.492(6)	H10	C10	C11	120.2
C17	H17	0.93	C10	C11	H11	119.5
C17	C18	1.384(6)	C10	C11	C12	120.9(6)
C18	H18	0.93	H11	C11	C12	119.6
C20	H20A	0.96	C7	C12	C11	119.8(5)
C20	H20B	0.96	C7	C12	H12	120.1
C20	H20C	0.959	C11	C12	H12	120.1
			P1	C13	C14	120.0(3)
			P1	C13	C18	121.1(4)
			C14	C13	C18	118.9(4)
			C13	C14	H14	119.7
			C13	C14	C15	120.5(4)
			H14	C14	C15	119.8
			C14	C15	H15	119.8
			C14	C15	C16	120.4(4)

H15	C15	C16	119.8
C15	C16	C17	119.7(4)
C15	C16	C19	117.8(4)
C17	C16	C19	122.5(4)
C16	C17	H17	120.1
C16	C17	C18	119.8(4)
H17	C17	C18	120.1
C13	C18	C17	120.7(5)
C13	C18	H18	119.7
C17	C18	H18	119.7
O1	C19	O2	124.1(5)
O1	C19	C16	124.1(4)
O2	C19	C16	111.8(4)
O2	C20	H20A	109.5
O2	C20	H20B	109.4
O2	C20	H20C	109.5
H20A	C20	H20B	109.4
H20A	C20	H20C	109.5
H20B	C20	H20C	109.5

Table 5S. Bond length (Å) and angles (°) for compound **4**.

Atom1	Atom2	Length	Atom1	Atom2	Atom3	Angle
Au1	Cl1	2.858	Cl1	Au1	P1	100.44
Au1	P1	2.307	Cl1	Au1	P1	100.44
Au1	P1	2.307	P1	Au1	P1	159.12
P1	C1	1.825(6)	Au1	P1	C1	116.4
P1	C8	1.818(7)	Au1	P1	C8	110.4
P1	C14	1.809(5)	Au1	P1	C14	110.9
O1	H1	0.821	C1	P1	C8	106.0(2)
O1	C7	1.324(9)	C1	P1	C14	106.1(2)
O2	C7	1.206(7)	C8	P1	C14	106.5(2)
C1	C2	1.39(1)	H1	O1	C7	109.5
C1	C6	1.401(7)	P1	C1	C2	119.0(4)
C2	H2	0.93	P1	C1	C6	121.6(4)
C2	C3	1.38(1)	C2	C1	C6	119.4(5)
C3	H3	0.929	C1	C2	H2	120.3
C3	C4	1.368(8)	C1	C2	C3	119.4(6)
C4	C5	1.38(1)	H2	C2	C3	120.3
C4	C7	1.501(9)	C2	C3	H3	119.1
C5	H5	0.931	C2	C3	C4	121.7(7)
C5	C6	1.385(8)	H3	C3	C4	119.2
C6	H6	0.93	C3	C4	C5	119.1(6)
C8	C9	1.371(9)	C3	C4	C7	122.1(6)
C8	C13	1.384(7)	C5	C4	C7	118.8(6)
C9	H9	0.93	C4	C5	H5	119.6
C9	C10	1.38(1)	C4	C5	C6	120.9(6)
C10	H10	0.93	H5	C5	C6	119.4

C10	C11	1.374(9)	C1	C6	C5	119.4(6)
C11	H11	0.93	C1	C6	H6	120.3
C11	C12	1.36(1)	C5	C6	H6	120.3
C12	H12	0.93	O1	C7	O2	124.5(7)
C12	C13	1.39(1)	O1	C7	C4	112.2(6)
C13	H13	0.93	O2	C7	C4	123.2(6)
C14	C15	1.390(9)	P1	C8	C9	118.6(4)
C14	C19	1.390(8)	P1	C8	C13	121.8(5)
C15	H15	0.93	C9	C8	C13	119.5(6)
C15	C16	1.40(1)	C8	C9	H9	119.6
C16	H16	0.931	C8	C9	C10	120.8(6)
C16	C17	1.36(1)	H9	C9	C10	119.6
C17	H17	0.93	C9	C10	H10	120.4
C17	C18	1.36(1)	C9	C10	C11	119.4(7)
C18	H18	0.93	H10	C10	C11	120.2
C18	C19	1.406(9)	C10	C11	H11	119.8
C19	H19	0.93	C10	C11	C12	120.5(7)
P1	C1	1.825(6)	H11	C11	C12	119.7
P1	C8	1.818(7)	C11	C12	H12	119.9
P1	C14	1.809(5)	C11	C12	C13	120.2(7)
O1	H1	0.821	H12	C12	C13	119.9
O1	C7	1.324(9)	C8	C13	C12	119.5(7)
O2	C7	1.206(7)	C8	C13	H13	120.3
C1	C2	1.39(1)	C12	C13	H13	120.2
C1	C6	1.401(7)	P1	C14	C15	122.9(4)
C2	H2	0.93	P1	C14	C19	118.1(4)
C2	C3	1.38(1)	C15	C14	C19	118.9(5)
C3	H3	0.929	C14	C15	H15	120.2
C3	C4	1.368(8)	C14	C15	C16	119.5(6)
C4	C5	1.38(1)	H15	C15	C16	120.3
C4	C7	1.501(9)	C15	C16	H16	119.4
C5	H5	0.931	C15	C16	C17	121.1(7)
C5	C6	1.385(8)	H16	C16	C17	119.4
C6	H6	0.93	C16	C17	H17	119.8
C8	C9	1.371(9)	C16	C17	C18	120.3(7)
C8	C13	1.384(7)	H17	C17	C18	119.8
C9	H9	0.93	C17	C18	H18	120
C9	C10	1.38(1)	C17	C18	C19	120.1(7)
C10	H10	0.93	H18	C18	C19	119.9
C10	C11	1.374(9)	C14	C19	C18	120.1(6)
C11	H11	0.93	C14	C19	H19	119.9
C11	C12	1.36(1)	C18	C19	H19	120
C12	H12	0.93	Au1	P1	C1	116.4
C12	C13	1.39(1)	Au1	P1	C8	110.4
C13	H13	0.93	Au1	P1	C14	110.9
C14	C15	1.390(9)	C1	P1	C8	106.0(2)
C14	C19	1.390(8)	C1	P1	C14	106.1(2)
C15	H15	0.93	C8	P1	C14	106.5(2)

C15	C16	1.40(1)	H1	O1	C7	109.5
C16	H16	0.931	P1	C1	C2	119.0(4)
C16	C17	1.36(1)	P1	C1	C6	121.6(4)
C17	H17	0.93	C2	C1	C6	119.4(5)
C17	C18	1.36(1)	C1	C2	H2	120.3
C18	H18	0.93	C1	C2	C3	119.4(6)
C18	C19	1.406(9)	H2	C2	C3	120.3
C19	H19	0.93	C2	C3	H3	119.1
O3	H3A	0.819	C2	C3	C4	121.7(7)
O3	C20	1.32(1)	H3	C3	C4	119.2
C20	H20A	0.96	C3	C4	C5	119.1(6)
C20	H20B	0.96	C3	C4	C7	122.1(6)
C20	H20C	0.96	C5	C4	C7	118.8(6)
			C4	C5	H5	119.6
			C4	C5	C6	120.9(6)
			H5	C5	C6	119.4
			C1	C6	C5	119.4(6)
			C1	C6	H6	120.3
			C5	C6	H6	120.3
			O1	C7	O2	124.5(7)
			O1	C7	C4	112.2(6)
			O2	C7	C4	123.2(6)
			P1	C8	C9	118.6(4)
			P1	C8	C13	121.8(5)
			C9	C8	C13	119.5(6)
			C8	C9	H9	119.6
			C8	C9	C10	120.8(6)
			H9	C9	C10	119.6
			C9	C10	H10	120.4
			C9	C10	C11	119.4(7)
			H10	C10	C11	120.2
			C10	C11	H11	119.8
			C10	C11	C12	120.5(7)
			H11	C11	C12	119.7
			C11	C12	H12	119.9
			C11	C12	C13	120.2(7)
			H12	C12	C13	119.9
			C8	C13	C12	119.5(7)
			C8	C13	H13	120.3
			C12	C13	H13	120.2
			P1	C14	C15	122.9(4)
			P1	C14	C19	118.1(4)
			C15	C14	C19	118.9(5)
			C14	C15	H15	120.2
			C14	C15	C16	119.5(6)
			H15	C15	C16	120.3
			C15	C16	H16	119.4
			C15	C16	C17	121.1(7)

H16	C16	C17	119.4
C16	C17	H17	119.8
C16	C17	C18	120.3(7)
H17	C17	C18	119.8
C17	C18	H18	120
C17	C18	C19	120.1(7)
H18	C18	C19	119.9
C14	C19	C18	120.1(6)
C14	C19	H19	119.9
C18	C19	H19	120
H3A	O3	C20	109.5
O3	C20	H20A	110
O3	C20	H20B	109
O3	C20	H20C	109
H20A	C20	H20B	110
H20A	C20	H20C	109
H20B	C20	H20C	109

Table 6S. Bond length (Å) and angles (°) for compound **7**.

Atom1	Atom2	Length	Atom1	Atom2	Atom3	Angle
C1	C2	1.372(9)	C2	C1	C6	118.5(6)
C1	C6	1.375(9)	C2	C1	P1	118.7(5)
C1	P1	1.803(6)	C6	C1	P1	122.8(5)
C2	H2	0.931	C1	C2	H2	120
C2	C3	1.38(1)	C1	C2	C3	120.2(7)
C3	H3	0.93	H2	C2	C3	119.8
C3	C4	1.37(1)	C2	C3	H3	120
C4	H4	0.93	C2	C3	C4	120.0(8)
C4	C5	1.34(1)	H3	C3	C4	120.1
C5	H5	0.931	C3	C4	H4	120.4
C5	C6	1.35(1)	C3	C4	C5	119.0(8)
C6	H6	0.93	H4	C4	C5	120.6
C7	C8	1.38(1)	C4	C5	H5	118.9
C7	C12	1.37(1)	C4	C5	C6	121.9(8)
C7	P1	1.821(7)	H5	C5	C6	119.2
C8	H8	0.93	C1	C6	C5	120.3(6)
C8	C9	1.39(1)	C1	C6	H6	119.9
C9	H9	0.93	C5	C6	H6	119.9
C9	C10	1.32(2)	C8	C7	C12	120.4(7)
C10	C11	1.36(2)	C8	C7	P1	122.4(6)
C10	C39	1.55(2)	C12	C7	P1	117.2(6)
C11	H11	0.93	C7	C8	H8	120.2
C11	C12	1.41(1)	C7	C8	C9	119.6(7)
C12	H12	0.93	H8	C8	C9	120.2
C13	C14	1.37(1)	C8	C9	H9	119.7
C13	C18	1.386(9)	C8	C9	C10	120.4(9)
C13	P1	1.801(7)	H9	C9	C10	120

C14	H14	0.93	C9	C10	C11	121(1)
C14	C15	1.38(1)	C9	C10	C39	122(1)
C15	H15	0.93	C11	C10	C39	117(1)
C15	C16	1.36(1)	C10	C11	H11	120
C16	H16	0.931	C10	C11	C12	120.5(9)
C16	C17	1.36(1)	H11	C11	C12	119.7
C17	H17	0.931	C7	C12	C11	117.7(8)
C17	C18	1.39(1)	C7	C12	H12	121.1
C18	H18	0.931	C11	C12	H12	121.1
C19	C20	1.370(7)	C14	C13	C18	118.8(7)
C19	C24	1.378(8)	C14	C13	P1	119.1(6)
C19	P2	1.815(6)	C18	C13	P1	122.1(6)
C20	H20	0.93	C13	C14	H14	119.7
C20	C21	1.390(9)	C13	C14	C15	120.7(7)
C21	H21	0.93	H14	C14	C15	119.6
C21	C22	1.373(8)	C14	C15	H15	120.1
C22	C23	1.386(8)	C14	C15	C16	119.8(9)
C22	C37	1.485(9)	H15	C15	C16	120.1
C23	H23	0.929	C15	C16	H16	120
C23	C24	1.37(1)	C15	C16	C17	120.7(9)
C24	H24	0.93	H16	C16	C17	120
C25	C26	1.37(1)	C16	C17	H17	120
C25	C30	1.37(1)	C16	C17	C18	119.8(9)
C25	P2	1.807(5)	H17	C17	C18	120.2
C26	H26	0.929	C13	C18	C17	120.1(7)
C26	C27	1.38(1)	C13	C18	H18	119.9
C27	H27	0.93	C17	C18	H18	120
C27	C28	1.37(1)	C20	C19	C24	119.0(5)
C28	H28	0.93	C20	C19	P2	123.2(4)
C28	C29	1.38(1)	C24	C19	P2	117.8(4)
C29	H29	0.93	C19	C20	H20	119.8
C29	C30	1.36(1)	C19	C20	C21	120.4(5)
C30	H30	0.93	H20	C20	C21	119.8
C31	C32	1.38(1)	C20	C21	H21	120.1
C31	C36	1.372(9)	C20	C21	C22	119.9(6)
C31	P2	1.798(5)	H21	C21	C22	120
C32	H32	0.93	C21	C22	C23	119.9(5)
C32	C33	1.36(1)	C21	C22	C37	118.1(5)
C33	H33	0.931	C23	C22	C37	122.0(5)
C33	C34	1.35(1)	C22	C23	H23	120.3
C34	H34	0.93	C22	C23	C24	119.2(6)
C34	C35	1.36(1)	H23	C23	C24	120.5
C35	H35	0.93	C19	C24	C23	121.5(6)
C35	C36	1.39(1)	C19	C24	H24	119.4
C36	H36	0.931	C23	C24	H24	119.1
C37	O1	1.188(8)	C26	C25	C30	119.0(7)
C37	O2	1.315(8)	C26	C25	P2	118.5(5)
C38	H38A	0.96	C30	C25	P2	122.4(5)

C38	H38B	0.959	C25	C26	H26	119.6
C38	H38C	0.96	C25	C26	C27	120.7(8)
C38	O2	1.45(1)	H26	C26	C27	119.7
C39	O3	1.25(2)	C26	C27	H27	120
C39	O4	1.23(2)	C26	C27	C28	119.2(9)
C40	H40A	0.96	H27	C27	C28	120
C40	H40B	0.96	C27	C28	H28	120
C40	H40C	0.96	C27	C28	C29	120.2(9)
C40	O4	1.50(1)	H28	C28	C29	120
Au	P1	2.303(2)	C28	C29	H29	120.3
Au	P2	2.303(2)	C28	C29	C30	119.4(8)
C41	C42	1.381(7)	H29	C29	C30	120.3
C41	C46	1.384(8)	C25	C30	C29	121.4(7)
C41	B	1.64(1)	C25	C30	H30	119.3
C42	H42	0.929	C29	C30	H30	119.3
C42	C43	1.39(1)	C32	C31	C36	118.8(6)
C43	C44	1.376(9)	C32	C31	P2	120.4(4)
C43	C47	1.47(1)	C36	C31	P2	120.8(4)
C44	H44	0.931	C31	C32	H32	119.8
C44	C45	1.37(1)	C31	C32	C33	120.4(7)
C45	C46	1.39(1)	H32	C32	C33	119.8
C45	C48	1.49(1)	C32	C33	H33	119.6
C46	H46	0.93	C32	C33	C34	121.0(7)
C47	F13	1.23(1)	H33	C33	C34	119.4
C47	F14	1.25(1)	C33	C34	H34	120.3
C47	F15	1.27(1)	C33	C34	C35	119.5(7)
C48	F10	1.26(1)	H34	C34	C35	120.2
C48	F11	1.38(1)	C34	C35	H35	119.8
C48	F12	1.29(1)	C34	C35	C36	120.5(7)
C49	C50	1.417(9)	H35	C35	C36	119.7
C49	C54	1.374(7)	C31	C36	C35	119.9(6)
C49	B	1.640(9)	C31	C36	H36	120.1
C50	H50	0.929	C35	C36	H36	120
C50	C51	1.38(1)	C22	C37	O1	123.7(6)
C51	C52	1.37(1)	C22	C37	O2	112.5(6)
C51	C56	1.49(2)	O1	C37	O2	123.9(7)
C52	H52	0.93	H38A	C38	H38B	109.6
C52	C53	1.36(1)	H38A	C38	H38C	109.5
C53	C54	1.401(9)	H38A	C38	O2	109.4
C53	C55	1.48(1)	H38B	C38	H38C	109.5
C54	H54	0.93	H38B	C38	O2	109.4
C55	F16	1.31(1)	H38C	C38	O2	109.4
C55	F17	1.32(1)	C10	C39	O3	115(1)
C55	F18	1.32(1)	C10	C39	O4	114(1)
C56	F19	1.23(1)	O3	C39	O4	131(2)
C56	F20	1.24(1)	H40A	C40	H40B	109
C56	F21	1.28(2)	H40A	C40	H40C	109
C57	C58	1.397(8)	H40A	C40	O4	109

C57	C62	1.408(7)	H40B	C40	H40C	110
C57	B	1.638(8)	H40B	C40	O4	109
C58	H58	0.93	H40C	C40	O4	109
C58	C59	1.390(8)	P1	Au	P2	177.82(6)
C59	C60	1.37(1)	C37	O2	C38	115.4(6)
C59	C64	1.46(1)	C39	O4	C40	110(1)
C60	H60	0.931	C1	P1	C7	108.2(3)
C60	C61	1.37(1)	C1	P1	C13	105.8(3)
C61	C62	1.364(9)	C1	P1	Au	112.8(2)
C61	C63	1.46(1)	C7	P1	C13	105.4(3)
C62	H62	0.931	C7	P1	Au	110.2(2)
C63	F7	1.22(1)	C13	P1	Au	114.0(2)
C63	F8	1.21(1)	C19	P2	C25	106.1(3)
C63	F9	1.22(2)	C19	P2	C31	104.8(2)
C64	F4	1.23(1)	C19	P2	Au	109.4(2)
C64	F5	1.26(2)	C25	P2	C31	108.3(3)
C64	F6	1.26(1)	C25	P2	Au	112.3(2)
C65	C66	1.395(8)	C31	P2	Au	115.3(2)
C65	C70	1.384(9)	C42	C41	C46	115.6(5)
C65	B	1.645(8)	C42	C41	B	124.9(5)
C66	H66	0.93	C46	C41	B	119.5(5)
C66	C67	1.372(8)	C41	C42	H42	118.8
C67	C68	1.37(1)	C41	C42	C43	122.3(5)
C67	C71	1.46(1)	H42	C42	C43	118.8
C68	H68	0.929	C42	C43	C44	120.0(6)
C68	C69	1.37(1)	C42	C43	C47	121.0(6)
C69	C70	1.392(9)	C44	C43	C47	119.0(7)
C69	C72	1.55(2)	C43	C44	H44	120.2
C70	H70	0.93	C43	C44	C45	119.5(7)
C71	F1	1.29(1)	H44	C44	C45	120.3
C71	F2	1.35(2)	C44	C45	C46	119.3(6)
C71	F3	1.19(1)	C44	C45	C48	121.2(7)
C72	F22	1.23(2)	C46	C45	C48	119.5(7)
C72	F23	1.34(3)	C41	C46	C45	123.3(6)
C72	F24	1.16(2)	C41	C46	H46	118.4
			C45	C46	H46	118.3
			C43	C47	F13	115.3(8)
			C43	C47	F14	116.0(8)
			C43	C47	F15	114.8(8)
			F13	C47	F14	105.0(9)
			F13	C47	F15	101.4(9)
			F14	C47	F15	102.5(8)
			C45	C48	F10	115.9(8)
			C45	C48	F11	109.5(8)
			C45	C48	F12	114.3(8)
			F10	C48	F11	103.1(8)
			F10	C48	F12	111.1(9)
			F11	C48	F12	101.2(8)

C50	C49	C54	114.1(5)
C50	C49	B	120.3(5)
C54	C49	B	125.6(5)
C49	C50	H50	118.6
C49	C50	C51	122.7(6)
H50	C50	C51	118.7
C50	C51	C52	120.7(7)
C50	C51	C56	118.7(7)
C52	C51	C56	120.6(7)
C51	C52	H52	120.4
C51	C52	C53	119.2(7)
H52	C52	C53	120.4
C52	C53	C54	119.7(6)
C52	C53	C55	119.7(6)
C54	C53	C55	120.6(6)
C49	C54	C53	123.7(5)
C49	C54	H54	118.2
C53	C54	H54	118.1
C53	C55	F16	114.4(7)
C53	C55	F17	114.6(7)
C53	C55	F18	111.9(7)
F16	C55	F17	104.9(7)
F16	C55	F18	105.7(7)
F17	C55	F18	104.4(7)
C51	C56	F19	116(1)
C51	C56	F20	116(1)
C51	C56	F21	114(1)
F19	C56	F20	104(1)
F19	C56	F21	103(1)
F20	C56	F21	102(1)
C58	C57	C62	113.0(5)
C58	C57	B	121.0(5)
C62	C57	B	126.0(5)
C57	C58	H58	118.1
C57	C58	C59	123.8(6)
H58	C58	C59	118
C58	C59	C60	120.0(6)
C58	C59	C64	120.5(7)
C60	C59	C64	119.5(7)
C59	C60	H60	120.9
C59	C60	C61	118.3(7)
H60	C60	C61	120.8
C60	C61	C62	120.9(6)
C60	C61	C63	120.1(7)
C62	C61	C63	119.0(7)
C57	C62	C61	123.9(6)
C57	C62	H62	118
C61	C62	H62	118.1

C61	C63	F7	117.4(9)
C61	C63	F8	117.4(9)
C61	C63	F9	116.3(9)
F7	C63	F8	104(1)
F7	C63	F9	100(1)
F8	C63	F9	98.7(9)
C59	C64	F4	114.7(8)
C59	C64	F5	114.8(8)
C59	C64	F6	116.5(8)
F4	C64	F5	103.9(9)
F4	C64	F6	104.4(8)
F5	C64	F6	100.8(8)
C66	C65	C70	113.7(5)
C66	C65	B	124.6(5)
C70	C65	B	121.7(5)
C65	C66	H66	117.5
C65	C66	C67	125.0(6)
H66	C66	C67	117.4
C66	C67	C68	119.1(6)
C66	C67	C71	119.9(7)
C68	C67	C71	121.0(7)
C67	C68	H68	120.7
C67	C68	C69	118.6(7)
H68	C68	C69	120.7
C68	C69	C70	121.0(8)
C68	C69	C72	118.6(9)
C70	C69	C72	120.3(9)
C65	C70	C69	122.4(7)
C65	C70	H70	118.8
C69	C70	H70	118.8
C67	C71	F1	113.4(9)
C67	C71	F2	111(1)
C67	C71	F3	120(1)
F1	C71	F2	95(1)
F1	C71	F3	112(1)
F2	C71	F3	102(1)
C69	C72	F22	115(1)
C69	C72	F23	103(1)
C69	C72	F24	113(1)
F22	C72	F23	111(2)
F22	C72	F24	106(2)
F23	C72	F24	109(2)
C41	B	C49	109.6(5)
C41	B	C57	106.7(5)
C41	B	C65	110.8(5)
C49	B	C57	110.7(5)
C49	B	C65	108.5(5)
C57	B	C65	110.5(5)

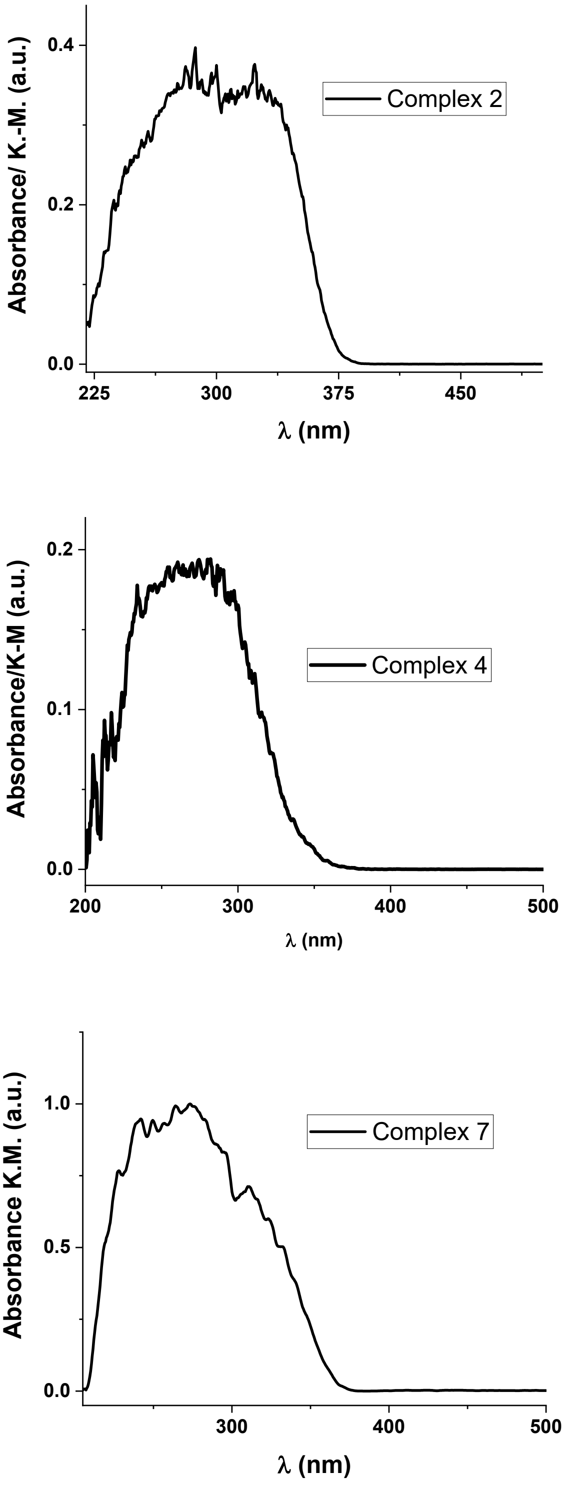

Figure 7S. UV-Vis spectra in solid state for complexes **2**, **4** and **7**.

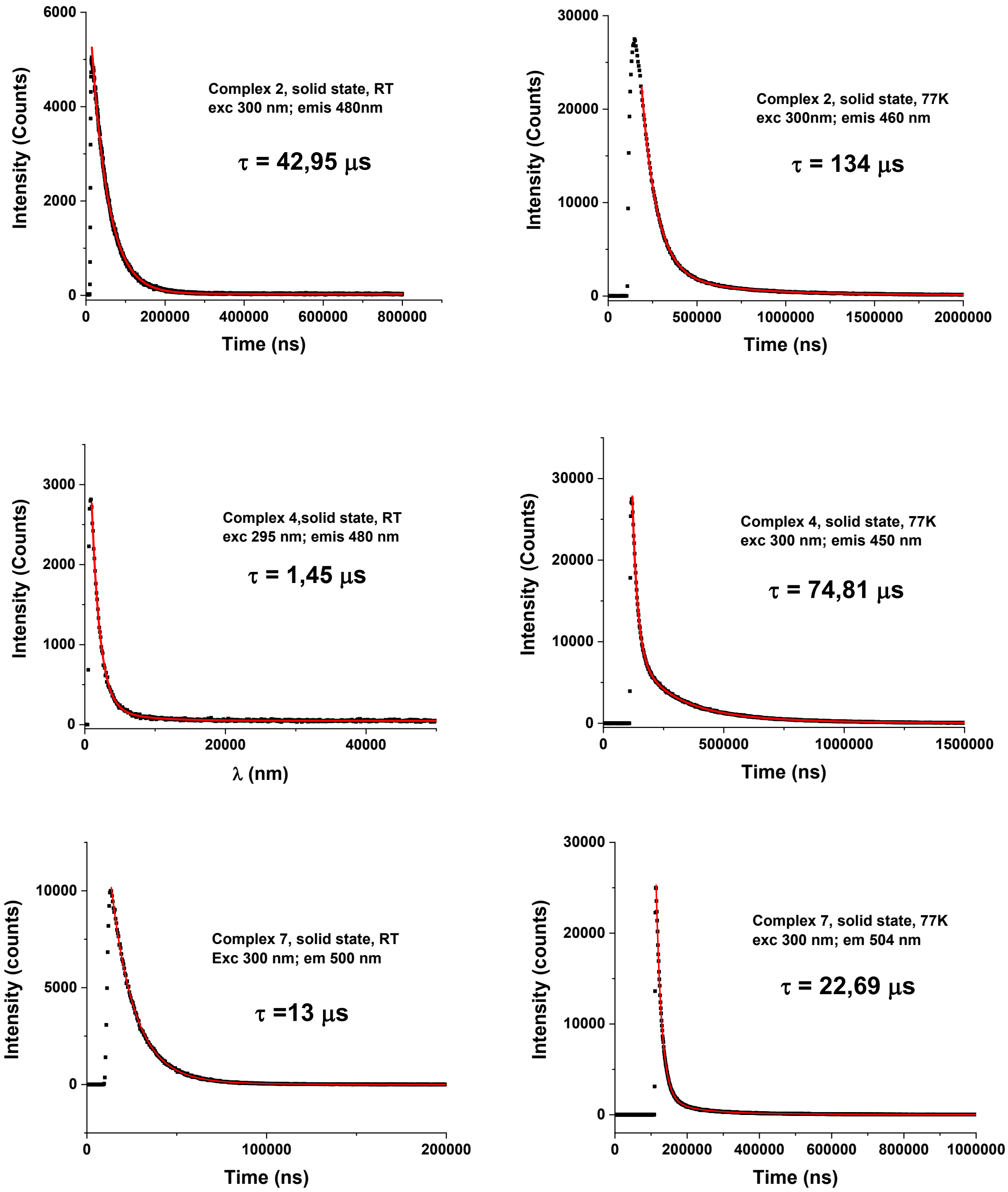

Figure 8S. Lifetime measurements for complexes **2**, **4** and **7** in solid state at RT and 77K.

Table 7S. XYZ COORDINATES FOR OPTIMIZED MODEL SYSTEMS

Model 4a in the ground state S_0
76

Au -0.014806 -0.005103 -5.359755
Cl -0.080361 -0.012528 -2.894902
P 0.000089 2.109958 -6.341278
O -0.011421 6.796236 -1.416233
H -0.127237 7.658723 -0.948672
O -0.817768 8.106537 -3.073595
C -0.148707 3.577047 -5.266171
C 0.234896 3.455973 -3.914943
H 0.555640 2.475768 -3.519762
C 0.157966 4.568400 -3.064710
H 0.449456 4.475986 -2.008086
C -0.310098 5.803421 -3.555557
C -0.709913 5.917946 -4.903964
H -1.081668 6.891399 -5.260368
C -0.633667 4.811532 -5.754720
H -0.955146 4.900675 -6.805022
C -0.416249 7.018813 -2.695005
C -1.409830 2.284784 -7.489597
C -2.704310 2.094168 -6.955163
H -2.825890 1.888631 -5.878085
C -3.828225 2.151314 -7.788858
H -4.832768 2.000645 -7.361259
C -3.673814 2.387297 -9.167010
H -4.558624 2.426889 -9.823256
C -2.389674 2.571373 -9.703403
H -2.262171 2.759382 -10.782290
C -1.259139 2.524783 -8.869513
H -0.254664 2.671896 -9.296701
C 1.480829 2.413430 -7.365104
C 2.167221 3.644652 -7.376220
H 1.821140 4.476170 -6.743099
C 3.299552 3.811881 -8.190043
H 3.833520 4.776227 -8.186294
C 3.751043 2.758941 -9.002182
H 4.641585 2.894427 -9.637573
C 3.071172 1.528754 -8.993760
H 3.422368 0.687996 -9.613714
C 1.948556 1.353484 -8.172829
H 1.433578 0.379306 -8.148539
P 0.003335 -2.109265 -6.362177
O -0.036519 -6.844893 -1.485392
H 0.077353 -7.711231 -1.024503
O 0.818303 -8.129875 -3.138287
C 0.151395 -3.584643 -5.298531
C -0.259745 -3.480447 -3.953916
H -0.598770 -2.507414 -3.555737
C -0.189372 -4.600659 -3.113481
H -0.502932 -4.521583 -2.062093
C 0.299515 -5.826535 -3.607190
C 0.727319 -5.923654 -4.948318
H 1.114790 -6.890146 -5.306930
C 0.657499 -4.809510 -5.789580
H 0.999817 -4.885426 -6.834238
C 0.398433 -7.049959 -2.757237
C 1.417228 -2.270700 -7.507414
C 2.710077 -2.080772 -6.969045
H 2.829062 -1.882831 -5.890311
C 3.835927 -2.129237 -7.800656
H 4.839231 -1.979357 -7.369861
C 3.685081 -2.355442 -9.180842
H 4.571381 -2.387954 -9.835472
C 2.402500 -2.538292 -9.721338
H 2.277605 -2.718390 -10.801877
C 1.270135 -2.500652 -8.889455
H 0.266961 -2.647299 -9.319809
C -1.472647 -2.409745 -7.393844
C -2.144908 -3.648273 -7.427731
H -1.791001 -4.486366 -6.807786
C -3.273170 -3.814401 -8.247312
H -3.796225 -4.784621 -8.261354
C -3.734681 -2.752830 -9.042468
H -4.622134 -2.887557 -9.682348
C -3.068918 -1.515363 -9.011238
H -3.428075 -0.668196 -9.617810
C -1.950146 -1.341520 -8.184703
H -1.446162 -0.362176 -8.142964

Model 4a in the excited state T_1
76

Au -0.058204 0.045557 -4.687883
Cl -0.136019 -0.252859 -2.371273
P -0.059142 1.808021 -6.267674
O -0.966512 7.162076 -2.173628
H -1.025976 8.104403 -1.885549
O -0.351896 8.284188 -4.043981
C -0.072111 3.375285 -5.370810
C -0.495250 3.443469 -4.018824
H -0.687220 2.515870 -3.452835
C -0.654640 4.678685 -3.388403
H -0.967392 4.723117 -2.334751
C -0.405740 5.882061 -4.093234
C 0.013950 5.814002 -5.446220
H 0.209420 6.758682 -5.977559
C 0.176966 4.585555 -6.079077
H 0.505445 4.551952 -7.130349
C -0.559578 7.218227 -3.477724
C -1.546562 1.759038 -7.287659
C -2.707912 1.138649 -6.764162
H -2.650574 0.587651 -5.810767
C -3.924367 1.219839 -7.448715

H -4.815409 0.725050 -7.030176
C -4.009125 1.919667 -8.666709
H -4.968911 1.983307 -9.204181
C -2.862652 2.546659 -9.188979
H -2.922208 3.107150 -10.136490
C -1.642186 2.479313 -8.505650
H -0.760294 2.998041 -8.913244
C 1.391820 1.892418 -7.347074
C 2.587252 2.426380 -6.811463
H 2.588344 2.848349 -5.793505
C 3.760615 2.435735 -7.577509
H 4.683237 2.865142 -7.154009
C 3.762471 1.900920 -8.876778
H 4.687488 1.905722 -9.475704
C 2.583434 1.347790 -9.405859
H 2.580237 0.913903 -10.418365
C 1.407574 1.337841 -8.645644
H 0.489011 0.903817 -9.065622
P 0.034603 -1.810425 -6.219401
O 1.027207 -7.260570 -2.281087
H 1.093140 -8.210182 -2.019503
O 0.366844 -8.332331 -4.165272
C 0.051392 -3.389863 -5.349317
C 0.509476 -3.492315 -4.011841
H 0.706067 -2.581510 -3.423584
C 0.687049 -4.744872 -3.421566
H 1.025899 -4.817002 -2.377537
C 0.420638 -5.929632 -4.151269
C -0.035218 -5.826085 -5.489461
H -0.245906 -6.755981 -6.040773
C -0.216954 -4.581321 -6.084264
H -0.573787 -4.520284 -7.124905
C 0.588682 -7.282039 -3.575732
C 1.536794 -1.723759 -7.211277
C 2.677128 -1.084658 -6.664080
H 2.592718 -0.537174 -5.710848
C 3.907440 -1.145049 -7.325031
H 4.782000 -0.636333 -6.889026
C 4.027019 -1.840692 -8.542745
H 4.997826 -1.886924 -9.061815
C 2.901393 -2.484911 -9.088771
H 2.988436 -3.042069 -10.036142
C 1.666767 -2.438788 -8.429833
H 0.801948 -2.972352 -8.854479
C -1.402900 -1.879873 -7.312598
C -2.608011 -2.404981 -6.789437
H -2.624569 -2.821203 -5.769220
C -3.770672 -2.411560 -7.571393
H -4.701936 -2.832216 -7.158360
C -3.750376 -1.884304 -8.873824
H -4.667179 -1.887227 -9.485300
C -2.561714 -1.341311 -9.390721
H -2.542990 -0.913025 -10.405370
C -1.396134 -1.332769 -8.614336
H -0.469506 -0.905088 -9.022322

Model 4b in the ground state S_0
264

Au 0.000000 0.000000 -6.334368
Cl 0.000000 0.000000 -3.546533
P -0.442656 2.245577 -6.727648
O 2.494432 5.754112 -1.752397
H 2.802786 6.498150 -1.080493
O 0.574489 6.955869 -1.851234
C -0.036706 3.329701 -5.330505
C 1.172495 3.071250 -4.652072
H 1.779878 2.191844 -4.922348
C 1.608874 3.925639 -3.635163
H 2.554659 3.721175 -3.113377
C 0.823019 5.036750 -3.266982
C -0.404119 5.272578 -3.917782
H -1.000736 6.143900 -3.605551
C -0.832665 4.429954 -4.951573
H -1.784236 4.628411 -5.469633
C 1.273470 6.010259 -2.213172
C -2.183474 2.545480 -7.168039
C -3.172074 1.689545 -6.640054
H -2.895989 0.863418 -5.962337
C -4.523345 1.905145 -6.951372
H -5.288578 1.233296 -6.530257
C -4.893590 2.966157 -7.793626
H -5.955413 3.129499 -8.040483
C -3.909606 3.816985 -8.325256
H -4.197153 4.648221 -8.989316
C -2.556710 3.609214 -8.016775
H -1.786119 4.271053 -8.443284
C 0.510895 2.937876 -8.121382
C 1.161071 4.185088 -8.038091
H 1.127501 4.762855 -7.101487
C 1.858361 4.688408 -9.148569
H 2.369460 5.661885 -9.074330
C 1.904780 3.956454 -10.345119
H 2.453837 4.354088 -11.214063
C 1.257043 2.711298 -10.431679
H 1.297570 2.130167 -11.367079
C 0.569508 2.200281 -9.323015
H 0.072284 1.217328 -9.378766
P 0.442656 -2.245577 -6.727648
O -2.494432 -5.754112 -1.752397
H -2.802786 -6.498150 -1.080493
O -0.574489 -6.955869 -1.851234
C 0.036706 -3.329701 -5.330505
C -1.172495 -3.071250 -4.652072
H -1.779878 -2.191844 -4.922348
C -1.608874 -3.925639 -3.635163
H -2.554659 -3.721175 -3.113377
C -0.823019 -5.036750 -3.266982

C 0.404119 -5.272578 -3.917782
H 1.000736 -6.143900 -3.605551
C 0.832665 -4.429954 -4.951573
H 1.784236 -4.628411 -5.469633
C -1.273470 -6.010259 -2.213172
C 2.183474 -2.545480 -7.168039
C 3.172074 -1.689545 -6.640054
H 2.895989 -0.863418 -5.962337
C 4.523345 -1.905145 -6.951372
H 5.288578 -1.233296 -6.530257
C 4.893590 -2.966157 -7.793626
H 5.955413 -3.129499 -8.040483
C 3.909606 -3.816985 -8.325256
H 4.197153 -4.648221 -8.989316
C 2.556710 -3.609214 -8.016775
H 1.786119 -4.271053 -8.443284
C -0.510895 -2.937876 -8.121382
C -1.161071 -4.185088 -8.038091
H -1.127501 -4.762855 -7.101487
C -1.858361 -4.688408 -9.148569
H -2.369460 -5.661885 -9.074330
C -1.904780 -3.956454 -10.345119
H -2.453837 -4.354088 -11.214063
C -1.257043 -2.711298 -10.431679
H -1.297570 -2.130167 -11.367079
C -0.569508 -2.200281 -9.323015
H -0.072284 -1.217328 -9.378766
O -2.960694 0.220247 -3.729850
H -1.954909 0.110051 -3.653941
C -3.280304 1.561363 -3.332012
H -3.495329 1.607675 -2.242380
H -4.189968 1.877537 -3.885872
H -2.447408 2.253031 -3.591092
Au -3.795646 -7.111127 3.328123
Cl -5.695678 -7.760218 1.496084
P -2.331392 -8.896338 3.540008
O -5.392528 -14.311412 0.793811
H -5.524296 -15.173815 0.330046
O -3.258481 -14.938966 0.386844
C -2.822363 -10.467091 2.751522
C -4.197639 -10.755765 2.630623
H -4.946223 -10.025060 2.975674
C -4.614771 -11.947028 2.023088
H -5.686967 -12.168292 1.918821
C -3.660316 -12.853551 1.518813
C -2.285823 -12.560084 1.631602
H -1.559681 -13.279783 1.222927
C -1.867019 -11.374946 2.244601
H -0.792797 -11.143664 2.319675
C -4.046267 -14.129628 0.846228
C -0.660758 -8.565192 2.897650
C -0.549215 -7.934807 1.640113
H -1.454792 -7.607436 1.099732
C 0.713252 -7.745492 1.058481
H 0.770904 -7.280136 0.061674
C 1.868701 -8.163873 1.739326
H 2.861563 -8.011285 1.285075
C 1.761747 -8.778680 2.998479
H 2.668102 -9.106775 3.533507
C 0.500600 -8.986438 3.578776
H 0.421225 -9.478831 4.560938
C -2.090375 -9.304976 5.300672
C -2.318456 -10.590965 5.825503
H -2.644644 -11.406740 5.161883
C -2.134058 -10.831394 7.197655
H -2.321606 -11.838773 7.603412
C -1.711802 -9.797207 8.046916
H -1.567329 -9.990263 9.122401
C -1.476064 -8.513116 7.523022
H -1.143412 -7.695287 8.183495
C -1.672229 -8.264133 6.159019
H -1.502659 -7.253602 5.751617
P -4.374560 -4.928648 3.906854
O -3.576594 -1.614549 -1.991130
H -3.448625 -0.860645 -2.698200
O -4.509635 0.052633 -0.769091
C -4.308037 -3.801290 2.484160
C -3.610101 -4.191079 1.323246
H -3.119980 -5.177459 1.280041
C -3.552620 -3.336890 0.213522
H -3.016029 -3.656085 -0.690919
C -4.188105 -2.082219 0.257845
C -4.888803 -1.693340 1.418034
H -5.373881 -0.705245 1.424738
C -4.958160 -2.547810 2.522264
H -5.524493 -2.248080 3.418966
C -4.115354 -1.103860 -0.882978
C -6.030615 -4.673631 4.617486
C -7.121042 -5.102706 3.832772
H -6.972012 -5.589280 2.856176
C -8.432765 -4.885889 4.273413
H -9.261434 -5.209447 3.623624
C -8.666001 -4.263581 5.511433
H -9.697750 -4.098547 5.863396
C -7.581009 -3.845673 6.300306
H -7.759608 -3.352469 7.269948
C -6.263149 -4.040355 5.855237
H -5.419436 -3.692014 6.470944
C -3.207483 -4.258307 5.137288
C -2.431329 -3.108748 4.893973
H -2.538454 -2.562972 3.943610
C -1.513249 -2.662523 5.859317
H -0.902739 -1.767783 5.655070
C -1.373141 -3.349050 7.075021
H -0.653509 -2.995000 7.830905
C -2.150321 -4.494747 7.324343
H -2.045654 -5.040574 8.276661
C -3.054209 -4.954431 6.357373
H -3.645041 -5.866714 6.545037
O 2.960694 -0.220247 -3.729850

H 1.954909 -0.110051 -3.653941
C 3.280304 -1.561363 -3.332012
H 3.495329 -1.607675 -2.242380
H 4.189968 -1.877537 -3.885872
H 2.447408 -2.253031 -3.591092
Au 3.795646 7.111127 3.328123
Cl 5.695678 7.760218 1.496084
P 2.331392 8.896338 3.540008
O 5.392528 14.311412 0.793811
H 5.524296 15.173815 0.330046
O 3.258481 14.938966 0.386844
C 2.822363 10.467091 2.751522
C 4.197639 10.755765 2.630623
H 4.946223 10.025060 2.975674
C 4.614771 11.947028 2.023088
H 5.686967 12.168292 1.918821
C 3.660316 12.853551 1.518813
C 2.285823 12.560084 1.631602
H 1.559681 13.279783 1.222927
C 1.867019 11.374946 2.244601
H 0.792797 11.143664 2.319675
C 4.046267 14.129628 0.846228
C 0.660758 8.565192 2.897650
C 0.549215 7.934807 1.640113
H 1.454792 7.607436 1.099732
C -0.713252 7.745492 1.058481
H -0.770904 7.280136 0.061674
C -1.868701 8.163873 1.739326
H -2.861563 8.011285 1.285075
C -1.761747 8.778680 2.998479
H -2.668102 9.106775 3.533507
C -0.500600 8.986438 3.578776
H -0.421225 9.478831 4.560938
C 2.090375 9.304976 5.300672
C 2.318456 10.590965 5.825503
H 2.644644 11.406740 5.161883
C 2.134058 10.831394 7.197655
H 2.321606 11.838773 7.603412
C 1.711802 9.797207 8.046916
H 1.567329 9.990263 9.122401
C 1.476064 8.513116 7.523022
H 1.143412 7.695287 8.183495
C 1.672229 8.264133 6.159019
H 1.502659 7.253602 5.751617
P 4.374560 4.928648 3.906854
O 3.576594 1.614549 -1.991130
H 3.448625 0.860645 -2.698200
O 4.509635 -0.052633 -0.769091
C 4.308037 3.801290 2.484160
C 3.610101 4.191079 1.323246
H 3.119980 5.177459 1.280041
C 3.552620 3.336890 0.213522
H 3.016029 3.656085 -0.690919
C 4.188105 2.082219 0.257845
C 4.888803 1.693340 1.418034
H 5.373881 0.705245 1.424738
C 4.958160 2.547810 2.522264
H 5.524493 2.248080 3.418966
C 4.115354 1.103860 -0.882978
C 6.030615 4.673631 4.617486
C 7.121042 5.102706 3.832772
H 6.972012 5.589280 2.856176
C 8.432765 4.885889 4.273413
H 9.261434 5.209447 3.623624
C 8.666001 4.263581 5.511433
H 9.697750 4.098547 5.863396
C 7.581009 3.845673 6.300306
H 7.759608 3.352469 7.269948
C 6.263149 4.040355 5.855237
H 5.419436 3.692014 6.470944
C 3.207483 4.258307 5.137288
C 2.431329 3.108748 4.893973
H 2.538454 2.562972 3.943610
C 1.513249 2.662523 5.859317
H 0.902739 1.767783 5.655070
C 1.373141 3.349050 7.075021
H 0.653509 2.995000 7.830905
C 2.150321 4.494747 7.324343
H 2.045654 5.040574 8.276661
C 3.054209 4.954431 6.357373
H 3.645041 5.866714 6.545037
O 3.234355 7.602122 -0.143381
H 4.124746 7.575768 0.358972
C 3.039677 8.902111 -0.697986
H 3.855947 9.169053 -1.408760
H 2.069338 8.877488 -1.236493
H 2.993024 9.671702 0.104818
O -8.103049 -5.789322 0.914961
H -7.434287 -6.523405 0.944051
C -7.499517 -4.685193 0.272766
H -7.371572 -4.837727 -0.829781
H -8.165876 -3.805837 0.416795
H -6.498010 -4.422498 0.695426
O -3.234355 -7.602122 -0.143381
H -4.124746 -7.575768 0.358972
C -3.039677 -8.902111 -0.697986
H -3.855947 -9.169053 -1.408760
H -2.069338 -8.877488 -1.236493
H -2.993024 -9.671702 0.104818
O 8.103049 5.789322 0.914961
H 7.434287 6.523405 0.944051
C 7.499517 4.685193 0.272766
H 7.371572 4.837727 -0.829781
H 8.165876 3.805837 0.416795
H 6.498010 4.422498 0.695426

Model 4b in the excited state T_1
264

Au 0.016949 -0.015855 -5.775590
Cl 0.071814 -0.038070 -3.055297
P -0.362610 2.239296 -6.221425
O 2.726296 6.146939 -1.634769
H 3.033767 6.966306 -1.072304
O 0.845874 7.388152 -1.883705
C 0.089165 3.411449 -4.925051
C 1.313453 3.200713 -4.246456
H 1.906721 2.293004 -4.444372
C 1.783225 4.146208 -3.330792
H 2.745216 3.982952 -2.824368
C 1.022223 5.303536 -3.051735
C -0.225808 5.482656 -3.688687
H -0.807485 6.386423 -3.448854
C -0.689737 4.551234 -4.621784
H -1.652749 4.712707 -5.131277
C 1.507815 6.378697 -2.132032
C -2.086156 2.577487 -6.691136
C -3.118566 1.791961 -6.137655
H -2.885748 0.976016 -5.431510
C -4.455351 2.062250 -6.468493
H -5.256561 1.444841 -6.031100
C -4.767940 3.107605 -7.353202
H -5.818768 3.314217 -7.614103
C -3.740270 3.887439 -7.910563
H -3.982158 4.706559 -8.607183
C -2.401525 3.624341 -7.584628
H -1.596201 4.231026 -8.028525
C 0.609754 2.745176 -7.683350
C 1.500455 3.834999 -7.658218
H 1.634254 4.415309 -6.732321
C 2.221175 4.177864 -8.814784
H 2.921199 5.028619 -8.786153
C 2.049757 3.444975 -9.999224
H 2.616659 3.717530 -10.904232
C 1.154407 2.360591 -10.029058
H 1.015119 1.783139 -10.957588
C 0.442982 2.006730 -8.875671
H -0.255245 1.152985 -8.893803
P 0.380687 -2.273037 -6.229076
O -2.732092 -6.141769 -1.630178
H -3.051396 -6.958189 -1.069013
O -0.870595 -7.409701 -1.887767
C -0.076979 -3.440892 -4.930847
C -1.297320 -3.217604 -4.249639
H -1.882059 -2.304089 -4.446594
C -1.773697 -4.157357 -3.331706
H -2.732157 -3.984300 -2.821943

C -1.023458 -5.321718 -3.053874
C 0.220817 -5.513989 -3.693820
H 0.794179 -6.423260 -3.454702
C 0.691620 -4.587655 -4.628837
H 1.651793 -4.758741 -5.140627
C -1.519350 -6.390988 -2.132349
C 2.100949 -2.620411 -6.703363
C 3.138321 -1.834277 -6.160173
H 2.912395 -1.011910 -5.459485
C 4.472645 -2.109667 -6.496655
H 5.277365 -1.491002 -6.067671
C 4.777885 -3.161240 -7.376493
H 5.826796 -3.372113 -7.641641
C 3.745297 -3.941901 -7.923626
H 3.981447 -4.765649 -8.616743
C 2.408981 -3.673263 -7.592466
H 1.599741 -4.280131 -8.028964
C -0.597901 -2.774601 -7.688137
C -1.493711 -3.860246 -7.660745
H -1.627751 -4.440086 -6.734593
C -2.218903 -4.199809 -8.815455
H -2.922855 -5.047257 -8.784997
C -2.047130 -3.467734 -10.000372
H -2.617616 -3.737668 -10.903909
C -1.146749 -2.387585 -10.032532
H -1.007157 -1.810795 -10.961426
C -0.430639 -2.037104 -8.881032
H 0.271741 -1.186806 -8.901151
O -2.910641 0.226307 -3.243600
H -1.909017 0.126293 -3.165093
C -3.259405 1.538661 -2.778577
H -3.440217 1.530825 -1.681317
H -4.195897 1.845744 -3.291764
H -2.458021 2.267894 -3.034608
Au -3.801864 -7.747308 3.233619
Cl -5.858162 -8.062745 1.597003
P -2.546706 -9.722412 3.137197
O -6.048576 -14.432169 -0.270204
H -6.234303 -15.198879 -0.863629
O -3.971187 -15.095059 -0.887205
C -3.167973 -11.098649 2.161176
C -4.568933 -11.304680 2.068561
H -5.260360 -10.616227 2.579419
C -5.077037 -12.351104 1.297498
H -6.163849 -12.500564 1.218422
C -4.200668 -13.214120 0.592436
C -2.801715 -13.004481 0.685697
H -2.135596 -13.682329 0.129616
C -2.288714 -11.964045 1.458295
H -1.200290 -11.805193 1.513090
C -4.684506 -14.327068 -0.253254

C -0.857408 -9.398846 2.547182
C -0.712681 -8.714999 1.319807
H -1.603150 -8.371625 0.764612
C 0.564889 -8.498549 0.783960
H 0.645595 -7.983913 -0.186339
C 1.702278 -8.946877 1.477228
H 2.707413 -8.771244 1.059732
C 1.561182 -9.624123 2.700153
H 2.452144 -9.982503 3.241565
C 0.285008 -9.856895 3.236385
H 0.180943 -10.399606 4.188765
C -2.365653 -10.312629 4.854841
C -2.801917 -11.591812 5.248401
H -3.237885 -12.274613 4.502086
C -2.680042 -11.992082 6.589713
H -3.027562 -12.992952 6.893185
C -2.114760 -11.125424 7.538477
H -2.018359 -11.443970 8.589138
C -1.670552 -9.849640 7.145976
H -1.219947 -9.166938 7.885549
C -1.802894 -9.439805 5.812899
H -1.461692 -8.436769 5.506753
P -4.261050 -5.559287 3.952559
O -3.472646 -1.745520 -1.626642
H -3.363384 -0.939343 -2.271814
O -4.273661 -0.158349 -0.217508
C -4.189610 -4.321712 2.637325
C -3.499361 -4.614280 1.441048
H -3.038730 -5.605491 1.295094
C -3.416437 -3.655840 0.423254
H -2.890645 -3.899580 -0.509540
C -4.016947 -2.392233 0.591915
C -4.705257 -2.100626 1.788670
H -5.160564 -1.103835 1.895066
C -4.801955 -3.057117 2.801827
H -5.355476 -2.829551 3.726553
C -3.937361 -1.315558 -0.451580
C -5.838342 -5.278314 4.812366
C -7.019500 -5.387118 4.045973
H -6.994203 -5.606913 2.964734
C -8.265470 -5.180202 4.652400
H -9.173757 -5.258315 4.033706
C -8.346931 -4.877849 6.022325
H -9.329196 -4.721805 6.497847
C -7.172523 -4.768494 6.784809
H -7.228716 -4.520987 7.857431
C -5.918494 -4.963267 6.184618
H -5.003348 -4.858012 6.786957
C -2.949667 -5.126748 5.143567
C -2.129136 -3.994042 4.974807
H -2.287261 -3.324111 4.115607

C -1.104853 -3.725592 5.897701
H -0.461703 -2.842493 5.753810
C -0.898798 -4.576075 6.995886
H -0.093754 -4.361733 7.717263
C -1.717482 -5.706408 7.169839
H -1.559715 -6.380276 8.028007
C -2.730445 -5.988082 6.243344
H -3.354276 -6.888742 6.367954
O 3.056831 -0.210013 -3.280440
H 2.054007 -0.134018 -3.189708
C 3.443172 -1.512375 -2.817389
H 3.642126 -1.496402 -1.723318
H 4.377688 -1.799963 -3.345319
H 2.654374 -2.260330 -3.057222
Au 3.776377 7.759218 3.225373
Cl 5.824203 8.152555 1.599853
P 2.476487 9.707513 3.145311
O 5.875982 14.517886 -0.223426
H 6.045596 15.290728 -0.813693
O 3.786222 15.129892 -0.851092
C 3.066583 11.104908 2.181142
C 4.462481 11.347662 2.097965
H 5.168355 10.676387 2.611734
C 4.948086 12.409313 1.333553
H 6.031111 12.587357 1.262080
C 4.054277 13.251529 0.625239
C 2.660480 13.005592 0.709321
H 1.980527 13.668032 0.151417
C 2.169669 11.949883 1.475459
H 1.085296 11.763581 1.523639
C 4.514806 14.378359 -0.214738
C 0.794143 9.350643 2.555427
C 0.661794 8.667784 1.326114
H 1.558214 8.343864 0.768847
C -0.611849 8.427890 0.790785
H -0.683366 7.915245 -0.181264
C -1.757135 8.851631 1.486443
H -2.759021 8.657290 1.069449
C -1.628271 9.528038 2.711210
H -2.525605 9.867541 3.254238
C -0.356456 9.784081 3.247080
H -0.262133 10.325935 4.200953
C 2.286902 10.276339 4.869044
C 2.703203 11.558233 5.275455
H 3.126662 12.255704 4.535569
C 2.577135 11.942348 6.621065
H 2.909004 12.945428 6.934508
C 2.027128 11.056800 7.561411
H 1.927158 11.362759 8.615463
C 1.602491 9.778392 7.156086
H 1.163970 9.080925 7.889081

C 1.739275 9.384695 5.818530
H 1.413337 8.379668 5.502451
P 4.300721 5.580224 3.928388
O 3.578923 1.770007 -1.661748
H 3.485333 0.963264 -2.309383
O 4.440593 0.201959 -0.266992
C 4.260362 4.348713 2.606983
C 3.546180 4.623890 1.420438
H 3.050993 5.599925 1.285268
C 3.482040 3.667825 0.399196
H 2.937155 3.898173 -0.525954
C 4.125955 2.423865 0.554107
C 4.838225 2.149744 1.740947
H 5.326652 1.167745 1.837467
C 4.915704 3.104090 2.757747
H 5.487268 2.890339 3.674728
C 4.067696 1.349686 -0.493066
C 5.885210 5.340959 4.787077
C 7.063259 5.474263 4.019609
H 7.032104 5.685006 2.936332
C 8.313759 5.303448 4.628182
H 9.220584 5.400563 4.010009
C 8.402705 5.013103 6.000182
H 9.388485 4.884979 6.476782
C 7.231285 4.879170 6.763332
H 7.293519 4.640867 7.837707
C 5.973076 5.037914 6.161635
H 5.060912 4.913439 6.764813
C 3.001233 5.106599 5.117228
C 2.216597 3.949183 4.945483
H 2.396699 3.285306 4.085822
C 1.199593 3.647801 5.866109
H 0.584459 2.745306 5.719981
C 0.965252 4.489876 6.965082
H 0.165825 4.249772 7.684606
C 1.748007 5.644834 7.142000
H 1.567855 6.312364 8.000731
C 2.753354 5.959199 6.217715
H 3.348342 6.878940 6.344445
O 3.482843 8.228883 -0.305131
H 4.323441 8.191628 0.252377
C 3.478587 9.405068 -1.115257
H 4.329830 9.407856 -1.836385
H 2.521067 9.400109 -1.675822
H 3.532091 10.320343 -0.485672
O -7.872657 -5.713648 0.960441
H -7.369249 -6.563881 1.057967
C -7.134390 -4.880136 0.088870
H -7.056663 -5.301224 -0.945721
H -7.667511 -3.907192 0.022736
H -6.096961 -4.676764 0.452172

O -3.522200 -8.212053 -0.306915
H -4.362150 -8.154291 0.250388
C -3.547923 -9.382877 -1.124187
H -4.399123 -9.359760 -1.844972
H -2.590865 -9.398662 -1.685385
H -3.624197 -10.300323 -0.500273
O 7.854550 5.830278 0.925623
H 7.351921 6.679685 1.032504
C 7.132912 5.019886 0.018951
H 7.107439 5.449783 -1.014732
H 7.645429 4.035093 -0.033297
H 6.077353 4.838899 0.338609
